# Nitrogen status exerts dynamic control over phosphorus sensing and acquisition via PSR1 in colimited marine diatoms

**DOI:** 10.1126/sciadv.adw8260

**Published:** 2025-08-29

**Authors:** Ellen Harrison, Yasmin Meeda, Trupti Gaikwad, Glen Wheeler, Katherine Helliwell

**Affiliations:** ^1^ Biosciences, University of Exeter, Exeter, EX4 4QD, UK.; ^2^ Marine Biological Association, Citadel Hill, Plymouth, PL1 2PB, UK.

## Abstract

Nutrient availability controls phytoplankton growth in aquatic ecosystems globally. Phytoplankton frequently experience a limiting supply of multiple nutrients simultaneously (colimitation). Ocean warming is predicted to exacerbate marine nutrient limitation. Yet, how phytoplankton adapt their physiology and regulate responses to colimitation is poorly understood. Here, we show that when the crucial macronutrients nitrogen (N) and phosphorus (P) colimit growth of globally abundant phytoplankton, the diatoms, cellular resources are diverted to prioritize N uptake over P sensing and acquisition. Regulatory mechanisms for responding to fluctuating P supply, including Phosphate Starvation Response 1 (PSR1) and P-resupply sensing via Ca^2+^ signaling, are strictly nitrate dependent. Further, P-Ca^2+^ signaling is impaired in *psr1* mutants, suggesting that PSR1 is necessary for this response and coordinates adaptations to colimitation. Our study demonstrates a hierarchy of resource allocation during colimitation that points to N starvation mechanisms overriding those of P and could lead to N-P colimitation being masked in many marine ecosystems.

## Teaser

Diatoms exert reciprocal, fine-scale control of P acquisition by N availability, depending on the immediate needs of the cell.

## INTRODUCTION

Analysis of phytoplankton communities globally has demonstrated that irrespective of temperature and latitude, net phytoplankton growth can be improved by the addition of multiple nutrients, indicating that marine surface waters are regularly in a state of nutrient colimitation (*1*). Nitrogen (N) is a major resource constraining phytoplankton growth, particularly in the low latitude subtropical gyres, with colimitation by N and iron (Fe) frequently observed at the boundaries of these regions (*2*). On the other hand, N and phosphorous (P) colimitation can occur in the subtropical Atlantic (*3*) and eastern North Pacific subtropical gyres (*1*), the Mediterranean Sea (*4*), and in coastal areas (*5*). Anthropogenic activities are altering the types of nutrients found in riverine outputs, causing shifts from primarily N-limited coastal systems to P limitation (*6*–*8*). Furthermore, ocean warming is changing global ocean cycles that will cause enhanced stratification and reduce nutrient upwelling (*2*, *9*, *10*). Perturbation of nutrient patterns could modify the population dynamics of phytoplankton communities altering net primary production in the oceans (*11*).

Diatoms are abundant globally distributed phytoplankton (*12*) that contribute substantially to marine primary production, with roughly 40% of organic carbon exported to the deep ocean attributable to diatoms (*13*, *14*). Diatoms often dominate early in spring algal blooms, due to their ability to rapidly sense and respond to changes in nutrient availability (*15*, *16*). Coastal regions frequently experience nutrient inflow from land and oceanic processes including upwelling and mixing, leading to pulses of nutrients allowing diatoms to thrive (*16*). Colimitation (or costress) in coastal ecosystems is also apparent for different combinations of nutrients (*17*, *18*), including N and P (*19*). It is therefore crucial to understand how alterations to nutrient availability, including colimitation, affects the physiology of diatoms. However, most studies have focused on diatom responses to singular N or P limitation (*20*–*23*) or the comparison between the two individual nutrient stressors (*24*, *25*).

By definition, colimitation is a state in which two or more nutrients restrict the growth of phytoplankton simultaneously (*2*, *26*). How colimitation affects phytoplankton plasticity of independent nutrient acquisition strategies and resource allocation is poorly understood. Moreover, colimitation can occur in different forms and is complicated by processes acting at the level of the cell and the community as a whole (*1*, *2*). While colimitation is widespread in nature, evidence has often been gathered at the community level, leading to gaps in understanding of how nutrient colimitation acts at the species level or even single cells in natural environments (*27*). The Redfield ratio of 16:1:106 N:P:C is a basic tenet of aquatic biogeochemistry and suggests that the elemental stoichiometry of the ocean chemistry is the same as that of phytoplankton (*28*). The ratio between N:P of 16:1 has often been used as a threshold for defining whether N or P is the limiting nutrient (*2*, *28*, *29*). In reality, the N:P ratio of phytoplankton is highly plastic, allowing phytoplankton to survive periods of nutrient deficiency (*2*, *28*, *30*).

To cope with P limitation, diatoms remodel phospholipids (*31*, *32*) and up-regulate alkaline phosphatases (*22*, *23*, *33*–*35*) and phosphate transporters (*34*, *36*). Coordination of these processes is controlled by the Myb-like transcription factor PtPSR1 (*Phaeodactylum tricornutum* P Starvation Response 1) (*36*) and long intergenic non protein coding RNAs (*22*). In addition, P-limited diatoms use a Ca^2+^ signaling pathway, to sense and rapidly respond to phosphate resupply (*37*). Whereas PSR1-like transcription factors are conserved across diverse photosynthetic eukaryotes (*38*–*42*), P-Ca^2+^ signaling has so far not been observed in green algae (*Chlamydomonas reinhardtii*) or plants (*43*, *44*), suggesting that diatoms have evolved distinct nutrient-sensing mechanisms for thriving in pulsed nutrient regimes. Diatom P-Ca^2+^ signaling is necessary for coordinating enhancements in N uptake following P resupply, mediating rapid regulatory cross-talk between P status and N acquisition. Trypsin, widely recognized as a digestive enzyme in animals, also represses N acquisition and enhances P uptake in diatoms experiencing P limitation (*45*). Hence, sophisticated interplay between diatom P and N signaling processes tightly control cellular investment in N acquisition when P is scarce. This is distinct from green algae since N uptake is maintained irrespective of P starvation in *C. reinhardtii* (*46*). Moreover, while N-P colimitation affects physiology and lipid metabolism of this alga, how such changes are regulated and effects of colimitation on N and P sensing and uptake have not been examined (*46*). There is a need to better understand nutrient sensing and regulatory mechanisms of diverse photosynthetic eukaryotes, including in the face of nutrient costress. For instance, it is unknown how diatom P-Ca^2+^ signaling is regulated by P limitation, and whether PtPSR1 coordinates this response. Moreover, while P-Ca^2+^ signaling acts to initiate N acquisition in P limited diatoms, the extent of the cross-talk between P and N, including whether N availability exerts reciprocal control of P regulatory mechanisms, and the consequences of P and N colimitation in diatoms are unexplored. As N deprivation typically enhances expression of N transport and assimilation genes (*20*, *24*, *47*), how such processes would respond to both N and P colimitation remains an open question.

In the marine environment, natural populations of diazotrophic Trichodesmium showed increased nitrogenase abundance under Fe and P costress, compared to single nutrient stress (*48*), as has also been observed in culture work with *Crocosphaera watsonii* (*49*). This highlights the need for more exploration of nutrient states experienced by natural populations when designing laboratory experiments and to better understand the regulatory processes governing nutrient physiology. Certainly, culture experiments with the prymnesiophyte, *Phaeocystis antarctica* grown in low manganese (Mn) and Fe resulted in the greatest difference in protein expression changes relative to the metal-replete conditions, and compared to Fe or Mn limitation singularly, and restructuring of photosynthetic apparatus, comparable to protein signatures in natural populations in the Southern Ocean (*50*). Although these studies and others (*51*, *52*) highlight impacts of colimitation, they focus predominantly on micronutrients. There is a clear need to investigate how N and P colimitation alters marine phytoplankton physiology and growth dynamics. In this work, we use genetically encoded fluorescent reporters, combined with measurements of diatom physiology and growth, to untangle aspects P signaling and acquisition in relation to N and P colimitation and resupply scenarios, with high temporal resolution.

## RESULTS

### Defining N and P colimiting conditions for the model pennate diatom *P. tricornutum*

**Fig. 1.**
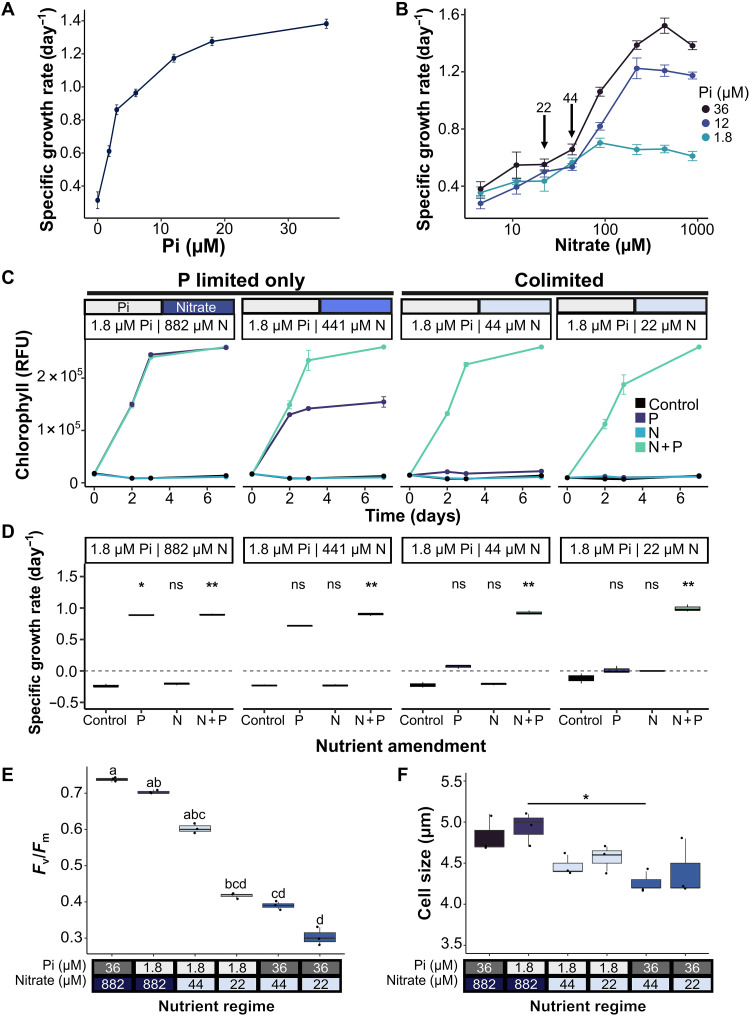
Determining P and N colimiting conditions for *P. tricornutum*. (**A**) Specific growth rate (day^−1^) of *P. tricornutum* (strain PtR1) grown on f/2 medium with different concentrations of phosphate (Pi): 0, 1.8, 3, 6, 12, 18, and 36 μM. The growth rate was calculated during exponential growth phase, between 2 and 4 days (*n* = 3, ±SEM). (**B**) As in (A), but cultures were grown on one of three Pi concentrations (1.8 μM in turquoise, 12 μM in navy blue, and 36 μM in black) but with various levels of nitrate, ranging from 0 to 882 μM (*n* = 3, ±SEM). (**C**) Nutrient amendment experiments with 4-day-old cultures grown in low Pi (1.8 μM) with varying nitrate levels between 22 and 882 μM. Cultures were grown with the addition of Pi (P, 36 μM), nitrate (N, 882 μM), nitrate and Pi (N + P), or with no supplementation (control) and changes in chlorophyll autofluorescence [relative fluorescence units (RFU)] over time shown as a proxy for growth (*n* = 3, ±SD). (**D**) The specific growth rate was calculated between days 0 and 3 after amendment of cultures shown in (C) (*n* = 3). Asterisks indicate results of Dunn post hoc test of addition treatments compared to the control: *P < 0.05 and **P < 0.01; “ns” indicates that the result was not significant. (**E**) Photosynthetic efficiency of photosystem II (*F*_v_/*F*_m_) of cells grown in various nutrient regimes for 4 days (*n* = 3). Letters indicate statistically significant groups, Dunn post hoc test, *P* ≤ 0.05. (**F**) Cell size (micrometers) of cells grown in various nutrient regimes for 4 days, measured using LUNA-FX7 (Logos Biosystems) (*n* = 3). Line indicates the only statistically significant comparison between high nitrate (882 μM) low phosphate (1.8 μM) and low nitrate (44 μM) high phosphate (36 μM), Tukey post hoc test, *P* ≤ 0.05.

*P. tricornutum* was grown in batch culture with phosphate concentrations ranging from 36 μM typical of standard f/2 medium (*53*, *54*) to zero phosphate. The specific growth rate (day^−1^) as measured between days 2 and 4 declined with decreasing initial phosphate levels (Fig. 1A). Three phosphate concentrations were subsequently taken forward including high (36 μM), intermediate (12 μM), and low (1.8 μM) phosphate, to examine nitrate-dependent growth. In high and intermediate phosphate treatments, specific growth rates began to decrease below 220 μM nitrate (Fig. 1B). However, in the low phosphate treatment, growth rates were comparable down to 88 μM nitrate, from which they declined with decreasing concentrations of nitrate, indicating that the cells were likely colimited. To confirm the colimiting conditions, we conducted nutrient amendment experiments with either single nutrient additions or simultaneous supplementation with both nitrate (882 μM) and phosphate (36 μM). A control treatment (“control”) was also conducted in which neither nutrient was added back. Growth stimulation with single nutrient amendment of phosphate was observed in cultures grown with initial concentrations of 1.8 μM phosphate and 882 μM or 441 μM nitrate, indicating growth limitation by phosphate only (Fig. 1, C and D). However, significant growth stimulation of 44 or 22 μM initial nitrate treatments was only achieved through supplementation with both nitrate and phosphate (Fig. 1D), indicating colimitation of cells by N and P. Total phosphate taken up per cell (that was exhausted from the medium) by day 4 was similar between P-limited only and low nitrate/low phosphate cells and substantially diminished compared to replete cells (fig. S1). Measuring the maximum quantum yield of photosystem II (*F*_v_/*F*_m_) for the different culture treatments revealed a reduction of P-limited compared to replete conditions, but a greater decrease in *F*_v_/*F*_m_ was seen in colimited cells (Fig. 1E). Inclusion of N-limited cultures to investigate further the distinct colimitation phenotype revealed that they had the lowest *F*_v_/*F*_m_ values of all. *P. tricornutum* cell size also varied significantly depending on the nutrient regime [analysis of variance (ANOVA), *P* = 0.015], with low phosphate high nitrate cells having the highest average cell size (4.93 μm, ±0.2 SD), followed by f/2 grown cells (4.83 μm, ±0.2 SD) (Fig. 1F). Cell size was lowest in the N-limited only cultures.

### N and P colimited diatom cells exhibit reduced capacity for P-Ca^2+^ signaling

**Fig. 2.**
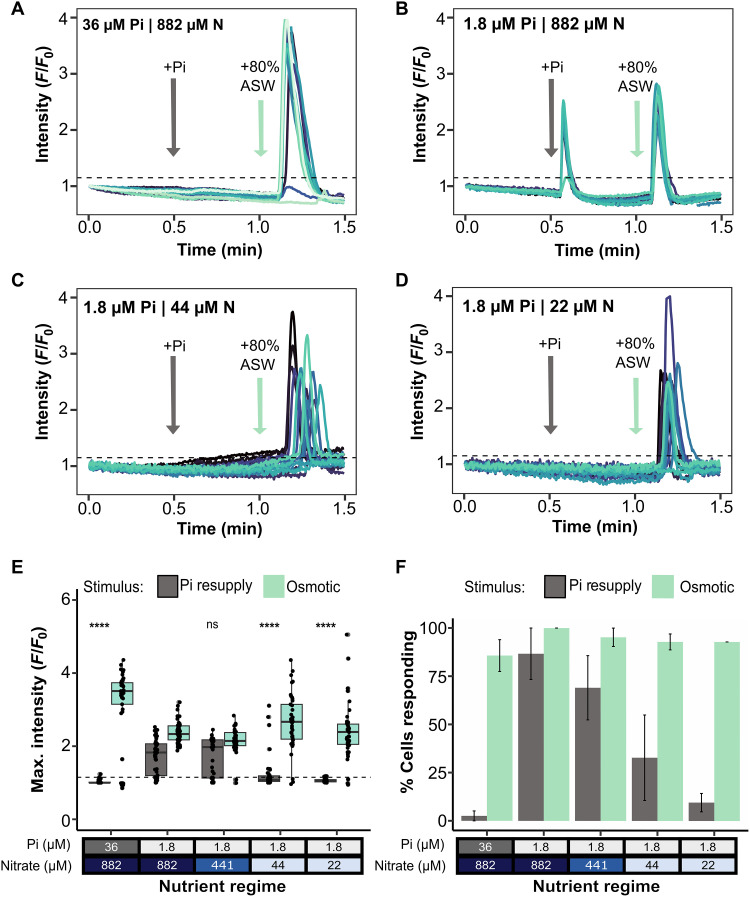
P and N colimited *P. tricornutum* cells grown in batch culture show a reduced capacity for P-Ca^2+^ signaling. (**A** to **D**) Example fluorescence traces of PtR1 cells grown in different nutrient regimes and exposed to either phosphate (Pi) resupply (36 μM Pi) stimulus or an 80% ASW osmotic shock, after 4 days of growth. Cells were deemed to have responded if the [Ca^2+^]_cyt_ fluorescence signal was above the *F*/*F*_0_ threshold of 1.15, indicated by the dashed horizontal line. (**E**) The maximal fluorescence (*F*/*F*_0_) of all cells analyzed to Pi resupply or osmotic shock, 1.15 threshold indicated by a dashed horizontal line. Asterisks indicate the Student’s *t* test result of Pi resupply responses in the different nutrient regimes compared to that of the P-limited only cells (1.8 μM Pi; 882 μM nitrate): ****P < 0.0001; ns indicates that the result was not significant. (**F**) Percentage of *P. tricornutum* PtR1 cells that respond to either stimulus, with at least 12 cells per replicate analyzed. Error bars indicate the SEM of three replicates.

We next tested the ability of cells grown under different nutrient regimes to sense phosphate resupply via P-Ca^2+^ signaling. We grew the transgenic *P. tricornutum* line PtR1 expressing the intensiometric fluorescent Ca^2+^ indicator, R-GECO1. Elevations in cytosolic Ca^2+^ in this strain are indicated by increases in R-GECO1 fluorescence intensity (*37*, *55*). Cells grown in replete, P limiting, or N and P colimiting conditions were perfused with artificial seawater (ASW) without phosphate for 0.5 min, before a 0.5-min exposure to 36 μM phosphate. A secondary exposure to hypo-osmotic shock was administered subsequently [to 80% ASW, diluted with double-deionised water (ddH_2_O)] to test the ability of cells for Ca^2+^ signaling more generally, as this treatment triggers robust cytosolic Ca^2+^ elevations in *P. tricornutum* (*56*). As observed previously (*37*), nutrient replete cells grown in standard f/2 medium for 4 days did not exhibit an elevation in cytosolic Ca^2+^ following phosphate resupply, although the same cells responded to hypo-osmotic shock (Fig. 2A). By comparison, cells grown in P-limiting conditions did respond to phosphate resupply (Fig. 2B). In contrast, colimited cells did not signal like P-limited only cells in response to phosphate resupply (Fig. 2, C to E), with only 33 and 9.5% responding in conditions with 1.8 μM Pi and either 44 or 22 μM nitrate, respectively (Fig. 2F). This was although more than 92% of cells responded to hypo-osmotic shock. To confirm that the observed reduction in the P-Ca^2+^ signaling response in N and P colimited cells was not a consequence of batch culture, cells were grown in the different nutrient regimes but semicontinuously (fig. S2A). Nutrient add-back experiments and culture growth rates confirmed N and P colimitation with 1.8 μM Pi and 44 μM (fig. S2, B and C). Moreover, like in batch culture, we observed a decline in the P-Ca^2+^ signaling ability (including both in the percentage of cells responding and the maximal *F*/*F*_0_) compared to those grown in low phosphate high nitrate (fig. S3). The diminished P-Ca^2+^ signaling response in colimited cells, which plays a key role in stimulating N acquisition following P resupply to P-limited cells (*56*), is particularly exciting as it suggests reciprocal regulation of this pathway by N availability.

### Rapid recovery of P-Ca^2+^ signaling within hours of nitrate resupply

**Fig. 3.**
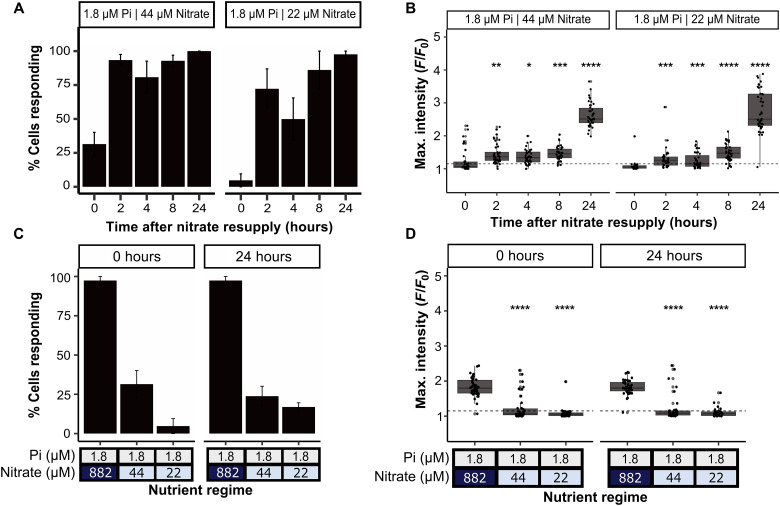
P-Ca^2+^ signaling response is restored within 2 hours following nitrate resupply to N and P colimited cells. (**A**) Time-course following nitrate resupply to 5-day-old N and P colimited cultures either grown in low Pi (1.8 μM) and 44 or 22 μM nitrate, showing the percentage of cells which respond to a Pi resupply (36 μM) stimulus, using a maximal fluorescence (*F*/*F*_0_) of 1.15 as the threshold for responding. Experiment performed on three sets of independently grown cultures per treatment, with at least 12 cells per replicate analyzed. Error bars show the SEM of three replicates. (**B**) Maximal fluorescence (*F*/*F*_0_) to Pi resupply of cells during the experimental time course. The horizontal line indicates 1.15 *F*/*F*_0_ threshold, and asterisks indicate the Student’s *t* test result compared to time point 0 (B) with *P* values described as follows: *P < 0.05, **P < 0.01, ***P < 0.001, and ****P < 0.0001; ns indicates that the result was not significant. (**C**) The control experiments, showing the percentage of cells that respond to Pi resupply when grown in low Pi (1.8 μM) high nitrate (882 μM) and the two colimited conditions at time point 0 and 24 hours later without nitrate resupply. (**D**) Corresponding maximal fluorescence (*F*/*F*_0_) to Pi resupply for the control experiments described in (C), the horizontal line indicates 1.15 *F*/*F*_0_ threshold, and asterisks indicate the Student’s *t* test result compared to low Pi high nitrate control *P* values described previously.

The capacity of cells for P-Ca^2+^ signaling decreases as a function of nitrate availability. As the control exerted by N availability is specific to the ability of cells to sense phosphate but not hypo-osmotic shock (Fig. 2), we hypothesized that the reduced P-Ca^2+^ signaling response was not a consequence solely of poor cell heath under colimiting nutrient conditions but rather a regulated process to optimize resource allocation when both P and N are scarce. To further investigate this, we tested whether, and over what timescale, colimited cells restore their ability for P-Ca^2+^ signaling, following resupply with nitrate. We took cells grown in batch culture with 1.8 μM and either 44 or 22 μM nitrate and added back 882 μM nitrate. We observed rapid restoration following amendment with nitrate of P-Ca^2+^ signaling ability, whereby the percentage of cells responding and the mean maximal intensity (*F*/*F*_0_) of the response increased within 2 hours for both the 44 and 22 μM treatments (Fig. 3, A and B). By contrast, colimited cells without nitrate add-back continued to have much lower responsiveness for P-Ca^2+^ signaling after 24 hours (Fig. 3, C and D), while nearly 100% of P-limited cells resupplied with phosphate showed the P-Ca^2+^ signaling response at time 0 and 24 hours. Similar trends were observed with cells grown in semicontinuous culture (fig. S4). Together, our data indicate that nitrate provision exerts dynamic control of the P-Ca^2+^ signaling response both under N and P nutrient colimitation, where the response is not only switched off but also following nitrate resupply when the capacity to sense phosphate resupply is rapidly recovered. Furthermore, as there is no restoration of growth 2 hours following nitrate resupply, these data demonstrate that differences observed in the P-Ca^2+^ signaling ability of colimited versus P-limited cells are not a consequence of subtle differences in growth rates between these treatments.

### N and P colimited *P. tricornutum* cells do not exhibit other characteristic P starvation–induced adaptations

**Fig. 4.**
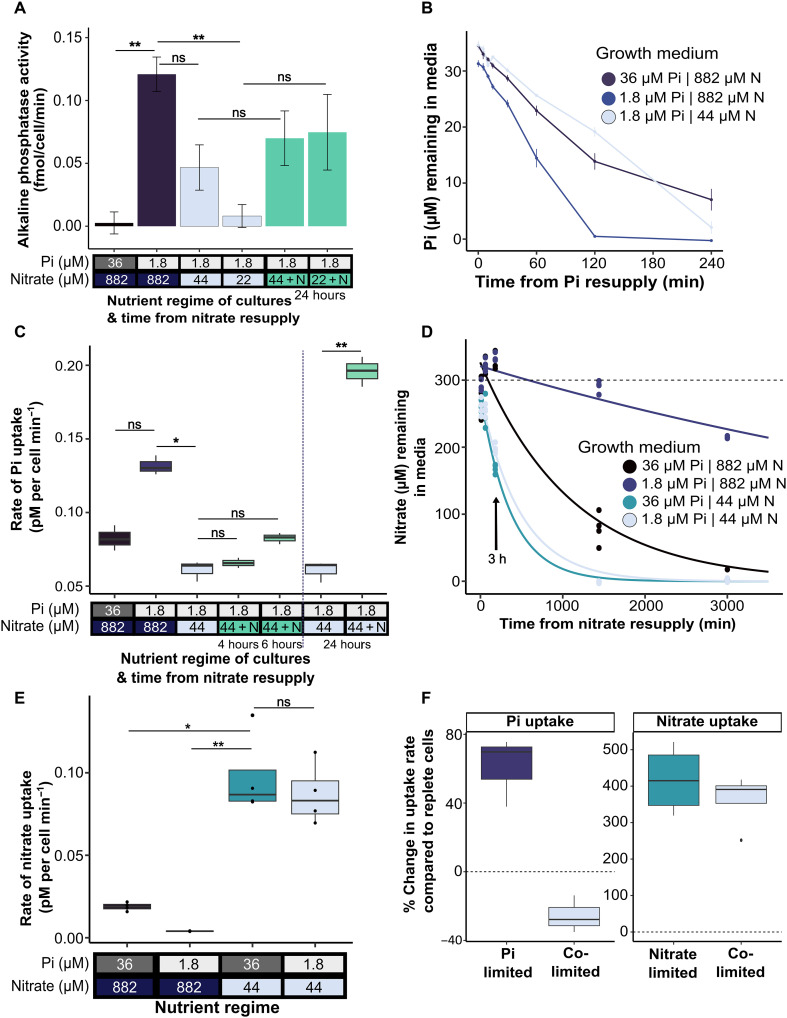
Dynamic control of P scavenging and acquisition by N availability in *P. tricornutum* and distinct colimitation physiologies. (**A**) Alkaline phosphatase activity (fmol/cell per min) of cells grown in f/2, low Pi (1.8 μM) high nitrate (882 μM), low Pi (1.8 μM) low nitrate (44 or 22 μM), or low Pi low nitrate resupplied with 882 μM nitrate 24 hours post-resupply (*n* = 3, ±SEM). Lines indicate statistical comparisons, and asterisks indicate the results of a Dunn post hoc test, *P* values described as follows: *P < 0.05 and **P < 0.01; ns indicates that the result was not significant. (**B**) Pi remaining in media fraction after Pi resupply (36 μM) to cells grown in various Pi and nitrate regimes (*n* = 3, ±SEM). (**C**) Rate of Pi uptake (picomolars per cell per minute) calculated between 30 and 120 min after Pi resupply (Fig. 4B). Pi uptake rates following nitrate resupply (882 μM) to low Pi (1.8 μM) low nitrate (44 μM) grown cells after 4, 6, and 24 hours, are also shown. Cells grown for 4 days and density normalized to 2 × 10^6^ cell/ml, before resupply *n* = 3, statistics displayed as in (A). Vertical dashed line to highlight measurements repeated 24 hours later for those samples. (**D**) Nitrate remaining in the media fraction after 300 μM nitrate resupply to cells grown in different nutrient regimes and cell density normalized ~8 × 10^6^ cell/ml, *n* = 4. Lines are fitted using nonlinear regression equation *f* = *a* × exp(−*b* × *x*). (**E**) Rate of nitrate uptake (picomolars per cell per minute) calculated between 1 and 3 hours for N-limited and colimited cells, 3 and 24 hours for replete cells, and 3 and 50 hours for P-limited cells, *n* = 4, statistics displayed as in (A). (**F**) Percent change in phosphate or nitrate uptake rates compared to replete cells for single-nutrient limitation and colimited cells.

In addition to exhibiting P-Ca^2+^ signaling, *P. tricornutum* up-regulates mechanisms to optimize P scavenging and acquisition during P limitation (*22*, *24*, *36*). However, how colimitation by N and P affects these processes is not known. We quantified cellular alkaline phosphatase activity in cells grown under the different nutrient regimes. Anticipated increases in enzyme activity were observed in P-limited compared to replete conditions (Fig. 4A). However, similar to the P-Ca^2+^ signaling response, alkaline phosphatase activity decreased substantially in N and P colimited conditions in batch (Fig. 4A) and semicontinuous (fig. S5, A and B) culture. Recovery of alkaline phosphatase activity was also observed following nitrate resupply. However, this occurred on a slower timescale compared to that seen for the P-Ca^2+^ signaling, with enzyme activity not fully restored even after 24 hours (Fig. 4A and fig. S5B).

Previously, we have observed rapid modulation of nitrate uptake rates in response to fluctuating phosphate supply in *P. tricornutum* (*37*). We therefore wanted to establish whether N availability exerts reciprocal control on phosphate uptake. By quantifying depletion of phosphate from the medium, we determined phosphate uptake capacity of cells grown in the different nutrient regimes in batch (Fig. 4, B and C) and semicontinuous (fig. S5C) conditions. Resupply of 36 μM phosphate to P-limited cells led to complete depletion of detectable external phosphate within ~2 hours (Fig. 4B). The timescale of this response far exceeded that of replete cells (Fig. 4B, dark blue). However, unexpectedly, the response was most slow in N and P colimited cells. Quantification of phosphate uptake rates revealed that cells grown in solely phosphate-limited conditions had a significantly higher uptake rate than P and N colimited cells (Dunn post hoc test, *P* = 0.014). We next examined over what timescale phosphate uptake capacity is restored following nitrate resupply. Increases in phosphate uptake rates were observable within 6 hours following nitrate resupply, but significant increases were seen only after 24 hours when rates far exceeded those (by 148%) even of solely P-limited cells (Fig. 4C). When grown semicontinuously, colimited cells also showed phosphate uptake rates that were substantially lower than in P-limited conditions, which increased significantly 24 hours following nitrate resupply (fig. S5C). Notably, protein/cell was also substantially diminished in colimited cells and did not increase significantly 6 hours following nitrate resupply (Dunn post hoc test, *P* = 0.361; fig. S5D).

As the colimited cultures showed the slowest phosphate uptake rates, we wanted to investigate nitrate uptake for comparison. In contrast to phosphate, we found that N-limited and colimited cells had a similar ability to take up nitrate from the medium, as compared to f/2 and P-limited cells (Fig. 4D). When the rate of nitrate uptake was calculated, there was not a significant difference between N-limited and colimited cells (Dunn post hoc test, *P* = 0.655), whereas f/2 and P-limited cells were significantly slower (Dunn post hoc test, *P* = 0.044 and 0.0012, respectively) (Fig. 4E). Calculating the percent change in uptake rates of phosphate versus nitrate in various nutrient limitation conditions compared to the replete cells, it can be seen that typical increases in phosphate uptake in P-limited cells are deprioritized in colimited cells, whereas N uptake is maintained irrespective of P and N colimitation (Fig. 4F).

### Nitrate-dependent control of transcriptional master regulator of P starvation responses PtPSR1

**Fig. 5.**
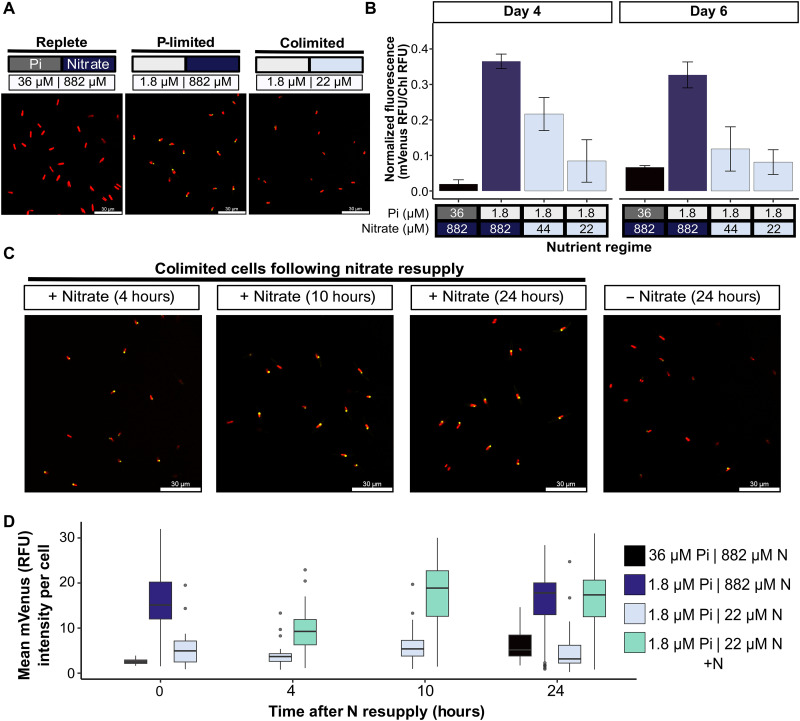
Expression of P starvation signaling master regulator PtPSR1 is governed by N availability. (**A**) Localization of PtPSR1-mVenus in *P. tricornutum* grown in f/2, low Pi (1.8 μM) high nitrate (882 μM) and low Pi (1.8 μM) low nitrate (22 μM), mVenus in yellow and chlorophyll in red. (**B**) Normalized fluorescence [mVenus RFU/chlorophyll (Chl) RFU] of *P. tricornutum* transformed with PtPSR1-mVenus reporter construct, grown in f/2 (36 μM PI, 882 μM nitrate) or low Pi (1.8 μM) with varying levels of nitrate (22-882 μM) after 4 days of growth (*n* = 4, ±SEM). (**C**) Localization of PtPSR1-mVenus in colimited cells over time with or without 882 μM nitrate resupply. Scale bars, 30 μm. (**D**) Mean mVenus fluorescence (RFU) per cell depending on nutrient regime and N resupply over 24 hours, at least eight cells per replicate analyzed with experiment performed on three sets of independently grown cultures per treatment (*n* ≥ 30).

As there was a reduction to both alkaline phosphatase activity and P uptake capabilities of *P. tricornutum* grown in N and P colimiting conditions, we investigated at what level these processes are regulated. The transcription factor *PtPSR1* is strongly up-regulated under P limitation and coordinates regulation of a plethora of *P. tricornutum* P starvation–induced responses (*36*). To investigate the role of PtPSR1 in coordinating the expression of P starvation–induced traits during colimitation, we generated a transgenic strain of *P. tricornutum* expressing PtPSR1 fluorescently tagged with mVenus, under the control of its native promoter region (Materials and Methods). Using epifluorescence microscopy, we observed mVenus fluorescence localized to the nucleus that was detectable in P-limited but not replete cells (Fig. 5A and fig. S6). As with the P-Ca^2+^ signaling response, expression of the PtPSR1 reporter construct was substantially reduced in N and P colimited cells. This was also observed measuring mVenus fluorescence normalized to chlorophyll fluorescence of bulk populations (Fig. 5B). Nitrate resupply to 1.8 μM phosphate, 22 μM nitrate grown cells revealed visibly increased PtPSR1 expression within just 4 hours (Fig. 5C). Quantifying PtPSR1-mVenus per cell (to avoid potential impacts of N and P limitation on cellular chlorophyll content) demonstrated conclusively that PtPSR1 expression was significantly reduced in colimited cells and rapidly significantly increased 4 hours after nitrate resupply, compared to the no nitrate resupply colimited control (Kruskal-Wallis test, *P* < 0.005) (Fig. 5D). Notably, the observed increase in PtPSR1 expression preceded any significant elevations in protein/cell in colimited cells following nitrate resupply (fig. S5D). Moreover, while a control strain expressing mVenus showed substantially lower expression in colimited (and P-limited) cells, unlike PtPSR1, fluorescence did not recover even 22 hours after nitrate addition (fig. S7). Thus, PtPSR1 expression under P limitation is tightly and rapidly regulated by N availability.

### *Ptpsr1* mutants show impaired P-Ca^2+^ signaling

**Fig. 6.**
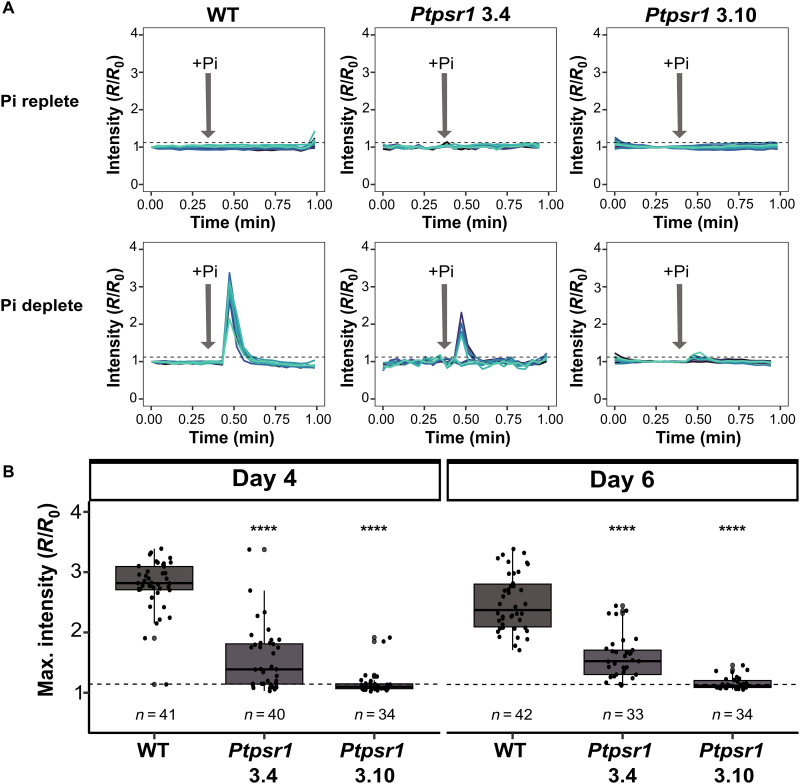
*Ptpsr1* mutants show impaired P-Ca^2+^ signaling. (**A**) Example traces of Ca^2+^ elevations in cells grown in replete (36 μM) or low (1.8 μM) Pi media for 4 days when resupplied with phosphate (Pi 36 μM, gray arrow). (**B**) Maximal fluorescence intensity after Pi resupply of all cells (*n*) tested on day 4 and day 6 for cells grown in low P medium only. Asterisks indicate the Student’s *t* test result compared to WT with *P* values described as follows: ****P < 0.001; ns indicates that the result was not significant. Experiments were performed on three sets of independently grown cultures per treatment, with at least nine cells per replicate analyzed. All three *P. tricornutum* strains were transformed with the R-GECO1–mTurquoise (RGMT) Ca^2+^ indicator, and fluorescence was normalized (*R*/*R*_0_) as described in the Materials and Methods.

We have demonstrated that while P-Ca^2+^ signaling is apparent in P-limited cells, it is substantially diminished in P and N colimited cells. In addition, expression of PtPSR1 is reduced under N and P colimitation. Both of these signaling responses are also rapidly restored over a similar timescale following nitrate resupply. However, it is unknown how the P-Ca^2+^ signaling pathway is controlled by P limitation. To investigate the possibility that this response is regulated by PtPSR1, we tested the ability of *Ptpsr1* mutants to exhibit phosphate-induced Ca^2+^ signals. *Ptpsr1* mutants (*Ptpsr1* 3.4 and *Ptpsr1* 3.10) previously generated and characterized by Sharma *et al.* (*36*) (fig. S8A) were transformed with the next generation of R-GECO1 probe R-GECO1–mTurquoise (*57*), which allows ratiometric imaging. Strains were grown in phosphate replete (36 μM) and low phosphate (1.8 μM) medium (fig. S8B). Both *Ptpsr1* 3.4 and *Ptpsr1* 3.10 mutants lack the Myb-DNA binding domain and nuclear localization signal and exhibit abolished or substantially impaired alkaline phosphatase activity and phosphate transporter expression under P limitation (*36*). Like wild type (WT), neither mutant showed a Ca^2+^ signaling response to phosphate when pregrown in P replete conditions (Fig. 6A). However, in contrast to WT, the P-limited *Ptpsr1* mutants showed little or no Ca^2+^ signaling when resupplied with phosphate. Both mutants had a significantly lower mean maximum fluorescence intensity ratio after phosphate resupply than WT on both days 4 and 6 (Student’s *t* test, *P* < 0.001). While this was higher in *Ptpsr1* 3.4 compared to *Ptpsr1* 3.10, there was still 45% decrease compared to the observed WT Ca^2+^ signal following phosphate resupply (Fig. 6B). Hence, our data demonstrate that normal induction of the P-Ca^2+^ signaling pathway under P limitation requires *PtPSR1*. As expression of PtPSR1 is substantially diminished under N and P colimitation, this evidence suggests that PtPSR1 is a key hub governing the distinct physiology observed during colimitation.

### *Thalassiosira pseudonana* exhibits nitrate-dependent regulation of P starvation–induced responses

**Fig. 7.**
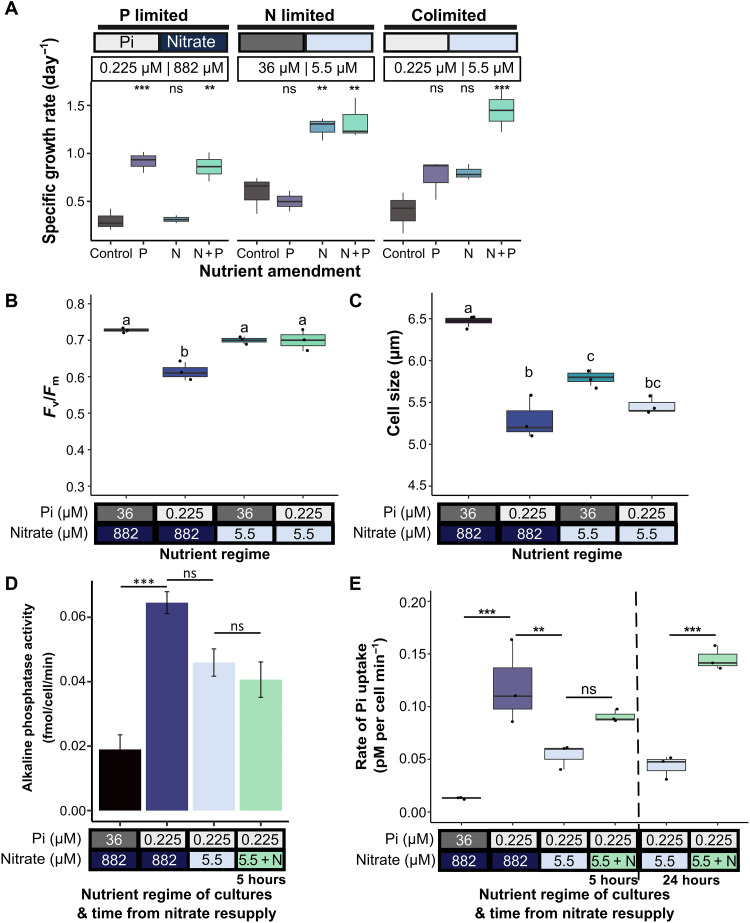
Regulation of P scavenging and phosphate uptake by N availability in the centric diatom *Thalassiosira pseudonana*. (**A**) Nutrient amendment experiments with cultures grown in a semicontinuous manner in low Pi (0.225 μM) medium with varying nitrate levels of 5.5 or 882 μM or in low nitrate (5.5 μM) and high phosphate (36 μM). Cultures were grown with the addition of Pi (P), nitrate (N), nitrate and Pi (N + P), or with no supplementation (control). The growth rate was calculated during exponential growth. Asterisks indicate the result of Tukey post hoc test only of an addition compared to the control: **P < 0.01 and ***P < 0.001; ns indicates that the result was not significant (*n* = 3). (**B**) Photosynthetic efficiency of photosystem II (*F*_v_/*F*_m_) of cells grown in various nutrient regimes semicontinuously (*n* = 3). Letters indicate statistically significant groups, Tukey post hoc test, *P* ≤ 0.001. (**C**) Cell size (micrometers) of cells grown in various nutrient regimes grown semicontinuously measured using LUNA-FX7 (Logos Biosystems) (*n* = 3). Letters indicate statistically significant groups, Tukey post hoc test, *P* ≤ 0.05. (**D**) Alkaline phosphatase activity (femtomoles per cell per minute) of cultures grown in N and Pi regimes after 10 days, N was resupplied to colimited cells, and alkaline phosphatase activity was measured after 5 hours (*n* = 3, ±SEM). Lines indicate statistical comparisons and asterisks the result of Tukey post hoc test, *P* values described as follows: **P < 0.01 and ***P < 0.001. (**E**) Rate of Pi uptake (picomolars per cell per minute) calculated between 60 and 150 min after Pi resupply (*n* = 3), statistics displayed as in (D). Vertical dashed line to highlight that uptake rate experiments were carried out again 24 hours later on those samples.

As diatoms represent one of the most diverse groups of microalgae (*12*, *58*), we lastly wanted to examine whether N and P colimitation causes similar impacts in other diatoms. *T. pseudonana* is an ecologically abundant centric diatom that exhibits characteristic responses to P limitation (*23*). To determine the nutrient concentrations necessary to cause P limitation and N and P colimitation, *T. pseudonana* was grown in batch culture with a range of nutrient concentrations. Lower levels of phosphate (i.e., 0.225 μM) were necessary to limit *T. pseudonana* growth (fig. S9A) as compared to *P. tricornutum* (Fig. 1A). Hence, to ensure that N and P were present in the same stoichiometry as in standard f/2 medium, and most of the above experiments for *P. tricornutum* (i.e., 1.8 μM phosphate and 44 μM nitrate treatment), we selected 0.225 μM phosphate and 5.5 μM nitrate for further experiments. The growth rate within this range of concentrations was more limited than the P-limited only treatment (i.e., 0.225 μM phosphate and 882 μM nitrate) when grown in batch conditions, indicative of colimitation (fig. S9B). This was confirmed in cells grown and acclimated to semicontinuous conditions (fig. S10), in which growth of *T. pseudonana* at these concentrations increased significantly only by addition of both nitrate and phosphate together (Fig. 7A). Notably, *F*_v_/*F*_m_ values of N-limited and colimited treatments did not differ significantly from replete conditions (Fig. 7B), whereas values for P-limited cells had a significantly lower *F*_v_/*F*_m_ than the other nutrient regimes (Tukey post hoc test, all comparisons *P* < 0.005). High *F*_v_/*F*_m_ values that do not decline under N limitation have previously been reported for *T. pseudonana* grown in steady state (chemostat), but not batch culture in which values reportedly declined with nutrient starvation (*59*). Hence, while we cannot conclude that our cultures grown semicontinuously were definitely in steady state, these data do suggest that our cells were exhibiting physiology more closely resembling cells in steady state than in batch (unbalanced) growth (*59*). Further physiological measurements revealed that cell size varied significantly depending on a nutrient regime (Fig. 7C; ANOVA, *P* < 0.001). Notably, alkaline phosphatase activity (Fig. 7D) and phosphate uptake rates (Fig. 7E) were both reduced in colimiting conditions compared to P limitation alone. However, the reductions in alkaline phosphatase activity were less pronounced compared to *P. tricornutum*: a decrease of 29% versus 69% in *T. pseudonana* (0.225 μM phosphate, 5.5 μM nitrate treatment) and *P. tricornutum* (1.8 μM phosphate, 44 μM nitrate treatment), respectively (Fig. 7D and fig. S5B). By comparison, phosphate uptake rates were reduced in colimited *T. pseudonana* cells, in a similar manner to *P. tricornutum*, i.e., 55% versus 50% decrease, respectively. Phosphate uptake rates also increased after 5 hours of nitrate resupply, with a significant difference detected after 24 hours (Tukey post hoc test, *P* < 0.005). Hence, N availability also affects the P scavenging and acquisition ability of *T. pseudonana*.

## DISCUSSION

Phytoplankton frequently experience scarce supply of multiple nutrients simultaneously, yet impacts of nutrient colimitation or costress on marine microbial physiology are poorly understood. We identify that despite severe cellular P demand, key physiological processes to maximize P acquisition and scavenging are substantially diminished and, in some cases, abolished in cells colimited by N and P (fig. S11). Our cumulative evidence indicates that this state is actively coordinated, rather than a general consequence of poor cell health caused by multinutrient scarcity. First, we identify that the control exerted by N availability on P starvation–induced responses occurs at the regulatory level, with expression of PtPSR1 strongly up-regulated under P limitation, but not N and P colimitation. Similarly, the ability of cells to sense phosphate resupply via Ca^2+^ signaling ceased under colimitation. Yet, both P signaling responses were rapidly restored within hours following nitrate resupply, preceding any significant increases in total protein/cell, as well as restoration of P scavenging and transport. Furthermore, unlike P-Ca^2+^ signaling, stress sensing of hypo-osmotic shock, which is frequently encountered in coastal ecosystems (*60*), was maintained irrespective of nutrient status. Lastly, and notably, the impacts of N and P colimitation on nutrient acquisition were not universally applied; N transport rates were equivalent in N limitation versus colimitation conditions and far exceeded those of the single P limitation treatment. By contrast, although P-limited cells can rapidly uptake phosphate, this ability is strongly diminished under colimitation. Hence, colimited diatoms prioritize investment in N acquisition over P. The maintenance of N uptake capacity during colimitation, which is typically up-regulated during N limitation and down-regulated in P-limited only cells (*37*, *45*), also points to N starvation regulatory mechanisms overriding those of P during colimitation. Together, our findings demonstrate that diatoms exhibit sophisticated, fine-scale, reciprocal regulation whereby the availability of one macronutrient influences uptake of the other, depending on the immediate needs of the cell.

It has long been recognized that the N:P ratio of phytoplankton cells is highly plastic (*28*). During P starvation, *Thalassiosira weissflogii* invests P almost exclusively in RNA (*61*). On the other hand, around 85% of N is dedicated to protein during N limitation (*62*). Phytoplankton can economize intracellular nutrient usage, including remodeling phospholipids to non-P forms (*31*). But our evidence indicates that diatoms also gate extracellular fluxes into the cell, prioritizing cellular resource acquisition toward N transport above P, hence autonomously regulating cellular stoichiometry. We are confident that the cells were colimited as N and P additions alone to low P low N cultures did not restore growth (Fig. 1C). Moreover, notably, the phosphate allocated per cell in colimited conditions was not significantly different from that of P-limited cells but substantially reduced compared to that of replete cells (fig. S1). The N:P input ratios in our low N low P treatments were also highly similar to the critical ratio, which marks the transition between N and P limitation, reported by Terry *et al*. (*63*) for *P. tricornutum* [i.e., 25 to 33 (*63*) versus 24.5 for the 44 μM nitrate, 1.8 μm phosphate treatment in our study]. The reduction in P acquisition in colimited cells suggests that allocation of cellular N to P transport, and even P sensing, in colimited cells is not advantageous. Evidently, N acquisition in colimited cells is necessary before there can be a response to P. Similarly, reductions in N acquisition in P-limited only cells (*37*, *45*) suggest that N transport is not beneficial when growth is in any case limited. However, P resupply subsequently triggers a signaling pathway that rapidly initiates N acquisition. As P resupply does not activate the Ca^2+^ signaling pathway in colimited cells, presumably because N uptake capacity is already up-regulated, this suggests that the primary role of the P-Ca^2+^ signaling pathway is to coordinate rapid enhancements in N uptake and assimilation following phosphate resupply to P-limited diatoms. These dynamics are likely constrained by costs and interdependencies associated with protein machinery for transporting and storing N versus P, as well as the transcripts encoding such proteins. Availability of membrane space may too be a factor when more than one nutrient is required (*64*, *65*).

Our study sheds important light on the molecular mechanisms mediating the distinct physiological adaptations observed under colimitation. PtPSR1 is a primary factor controlling up-regulation of P scavenging and uptake during P limitation in diatoms (*36*) as well as plants and green algae (*41*, *66*, *67*). *C. reinhardtii PSR1* overexpression lines and mutants have altered lipid accumulation in response to N starvation compared to WT (*42*). However, the role of PSR1 in regulating N and P cross-talk in algae, including under N and P colimitation, has received little attention. We demonstrate that *Ptpsr1* mutants show impaired P-Ca^2+^ signaling—indicating that PtPSR1 likely regulates machinery underlying this signaling response and demonstrating interplay between the P starvation signaling and P-Ca^2+^ signaling regulatory pathways. Decreases in expression/nuclear localization of PtPSR1 are mirrored by reduced P-Ca^2+^ signaling during colimitation. Further, both PtPSR1 and P-Ca^2+^ signaling capacity shows rapid responsiveness to N resupply. Thus, under colimitation, cells not only deprioritize resource allocation to P acquisition but also do not even activate mechanisms for P sensing. These results also suggest that PtPSR1 is a crucial factor governing the impairment of P-Ca^2+^ signaling and other P starvation–induced responses in colimited diatoms and thus a key hub for integrating N and P interactions. Direct molecular interactions between PSR1-like proteins and N signaling machinery mediate nitrate-dependent control of P starvation–induced responses in plants (*68*, *69*). In rice, the nitrate transceptor NRT1.1B that perceives nitrate on the plasma membrane physically interacts with and promotes the degradation of SPX4 (*68*). SPX4 binds the PSR1-like transcription factor PHR2 and represses its activity through preventing its translocation to the nucleus from the cytoplasm (*70*). Hence, nitrate-dependent degradation of SPX4 enables intersection of N and P signaling pathways. Under low N, SPX4 accumulates regardless of P levels, binding PHR2, thus preventing activation of P starvation–induced responses in the nucleus (*68*). Like plants, *P. tricornutum* encodes a candidate SPX repressor protein (PtSPX) (*71*), the cellular localization of which is unknown. However, mutants exhibit elevated expression of P transporters and alkaline phosphatases, consistent with it being a negative regulator of PtPSR1. Further work deducing protein interactions between PtPSR1 and PtSPX, including under N and P colimitation, may help better establish molecular mechanisms mediating N and P cross-talk in diatoms. Advances in understanding of N sensing and transport mechanisms are also necessary. Certainly, nitrate provision drives rapid transcriptional modulation in *P. tricornutum* within 15 min (*72*). *P. tricornutum* encodes six high-affinity *NRT2* and two low-affinity *NPF* (formally *NRT1*) genes for N transport (*73*, *74*), but whether they have dual roles for N transport and sensing, as in plants (*75*), and if restoration of intracellular N or perception of external levels govern the rapid regulation of P machinery remain to be elucidated.

Our results also have relevance in the environmental context. Alkaline phosphatase varies according to phosphate availability and has thus been used widely to diagnose global-scale patterns and temporal changes in P limitation in marine and freshwater ecosystems [e.g., (*76*–*79*)]. Here, we show that in conditions where both P and N are limiting, alkaline phosphatase activity is reduced up to ~75% in diatoms compared to P-limited only cells. Furthermore, our observation that phosphate uptake was strongly impaired in colimited diatoms relative to P-limited only cells, whereas N uptake rates were comparable between N deplete versus N and P colimiting conditions, suggests that N transport markers (e.g., NRT) may be more reliable at indicating N limitation irrespective of P availability. Hence, caution should be taken when using dual N and P markers that could potentially lead to an overestimation of sole N limitation when N and P are colimiting. Certainly, data from freshwater phytoplankton communities suggest that in low N and P environments, there are changes to the level of alkaline phosphatase activity detected (*80*). Alkaline phosphatase activity has been measured in regions of the (sub)tropical North Atlantic, where *Prochlorococcus* and *Synechococcus* populations exhibited N and P colimitation (*81*). Substantial increases in bulk alkaline phosphatase activity occurred following N amendment. This could be through enhanced P stress caused by relief from N limitation (*81*). Alternatively, since P stress should not become more pronounced in cells that are already P and N colimited, the enhanced alkaline phosphatase could reflect reallocation of N resources to P acquisition. Whether cyanobacteria dominating these oligotrophic open ocean habitats exhibit a hierarchy of resource allocation that minimizes cellular investment in P scavenging during colimitation like the diatoms studied here remains an important open question. Notably, another study comparing experimental nutrient amendment bioassays with metagenomic analysis of gene content of natural populations of *Procholorococcus* to infer nutrient limitation has revealed that P stress markers can be more pronounced even when N is the primary limiting nutrient (*1*, *78*). However, while assessing genomic attributes relating to nutrient acquisition no doubt provides crucial insights of the selective pressures (and specific nutrient stressors) shaping such communities over evolutionary timescales, it does not necessarily reflect the experienced nutrient limitation of phytoplankton (and their gene expression/physiology) at the point of sampling, unlike bioassays or enzyme activity. These considerations highlight the difficulties and uncertainties in diagnosing nutrient limitation patterns in situ, including the distinct insights and outputs gained from different diagnostics (e.g., ambient nutrient concentrations, growth responses, versus different biomarkers) and challenges in designing colimitation experiments to examine complex natural communities.

In the future, applying a molecular lens examining transcriptomic and proteomic shifts of diatoms under N and P colimitation will enable characterization of the proteins and transcripts mediating physiological outputs. This could help identify biomarkers that indicate P status irrespective of N availability, as well as colimitation, as has been done for micronutrient (Mn/Fe) costress (*50*), to facilitate autonomous sampling efforts across large oceanographic ranges (*79*). Further, the observed reciprocal integration of N and P signaling raises the question of how interdependencies arising from other limiting nutrients input into this regulatory module. Notably, PSR1 is involved in integrating P and sulfur starvation responses in *C. reinhardtii* (*41*, *82*). Cellular models of stoichiometry have also widened to include other elements and micronutrients including Fe, K, Ca, Zn, etc. (*2*, *83*). In low Zn, low Fe regions of the North Atlantic, Fe amendment experiments stimulated alkaline phosphatase activity (*81*), likely due to its role as a cofactor for alkaline phosphatase (*84*, *85*), i.e., biochemically dependent (or type III) colimitation (*26*). Additional cofactors for this enzyme (e.g., Zn, Mn, and Mg) (*35*, *86*) thus may too control P cycling. However, our study challenges current definitions of colimitation. N-P colimitation is typically defined as type I or “independent” colimitation, albeit within this definition acknowledging that there are always biochemical dependencies inherent to some extent but to a lesser degree relative to type II and III colimitation examples (*26*). Here, we quantify specifically which aspects of P metabolism are dependent on N availability and to what extent. In doing so, we provide a body of empirical evidence that multiple processes for P scavenging, uptake, and signaling are strongly N dependent. Hence, in support of modeling studies on chemostat cultures (*87*), N:P colimitation would be better categorized as biochemically dependent. This nuanced view goes hand in hand with work concluding microbes experience a continuum of colimitation states, rather than discrete categories of nutrient limitation (*27*). No doubt, predictions of changing nutrient patterns in the ocean (*2*, *9*, *10*), coupled with evidence of nutrient costress exacerbating harmful bloom toxicity (*18*), will bring understanding nutrient colimitation to the forefront of marine research.

## MATERIALS AND METHODS

### Strains and culturing

*P. tricornutum* CCAP1055/1 [Culture Collection of Algae and Protozoa, Scottish Association for Marine Science (SAMS), Oban UK] was used as the WT strain and background for all *P. tricornutum* strains in this work. In addition, *T. pseudonana* strain CCMP 1335 was used. *P. tricornutum* expressing R-GECO1 (PtR1) was generated as detailed in (*88*) and the G-GECO1-mApple expression line in (*89*). *P. tricornutum* and *T. pseudonana* were maintained in natural seawater with f/2 nutrients (*53*), except vitamins and silica for *P. tricornutum*. Cultures were illuminated with light (50-80 μmol m^−2^ s^−1^), with a 16:8-hour light:dark cycle at 18°C. For experiments, cultures were grown in ASW, using Tropic Marin Classic (Tropic Marin) sea salts (30 g/liter) with pH adjusted to 8.1 and sterile filtered, with f/2 nutrients added as before. For nutrient limitation experiments, cultures were grown with the standard trace element concentrations (also silica and vitamins for *T. pseudonana*), with phosphate and nitrate at reduced concentrations as described here in the text. For all experiments, cultures were inoculated at a cell density of 3 × 10^4^ cell/ml, using cultures grown for 4 to 5 days. Unless otherwise stated, experiments used 30-ml culture volumes in tissue culture flasks (catalog no. 83.3910.502, SARSTEDT) and were grown as described above.

To grow *P. tricornutum* for signaling experiments in batch conditions, cultures were grown in 6-well plates (catalog no. 83.3920, SARSTEDT) in a 6-ml volume. For the semicontinuous conditions, cells were inoculated into 24-well plates (catalog no. 83.3922, SARSTEDT) in a 2-ml volume and grown for 4 days with four replicates per nutrient regime [f/2, high nitrate (882 μM) low phosphate (1.8 μM) and the low nitrate low phosphate conditions (1.8 μM phosphate, 44 or 22 μM nitrate)], and a chlorophyll reading was taken using a CLARIOstar Plus plate reader (BMG Labtech) using (excitation/emission) 440 ± 9/680 ± 20 nm wavelengths. The cultures were then diluted to give a chlorophyll [relative fluorescence units (RFU)] reading of 4900 (the average of all day 1 readings) using ASW with the appropriate nutrient regime. This was repeated at 2- to 3-day intervals for 9 days with the signaling experiment carried out on day 15 on three of the biological replicates. Growth rates were monitored throughout the experiment. For the physiology experiments, including protein/cell experiments, cultures were inoculated in triplicate flasks (catalog no. 83.3912.502, SARSTEDT) with 200 ml of medium with the different nutrient regimes, except the 1.8 μM phosphate, 44 μM nitrate regime which had six replicates to allow for the N resupply protein per cell experiment. Cultures were grown for 4 days before diluting cultures every 2 to 3 days. For the first three dilution events the f/2 treatment, alongside the high nitrate low phosphate, and low nitrate high phosphate cultures were diluted to 1 × 10^6^ cell/ml, whereas colimited cultures were diluted to 0.65 × 10^6^ cell/ml. From day 12 onward, the single nutrient limited cultures were also diluted to 0.65 × 10^6^ cell/ml, and the other two nutrient treatments maintained the same dilution regime throughout. The five dilution events from day 12 onward were used to calculate the growth rates shown in fig. S5A. The experiment ran for 23 days, with phosphate uptake experiment on day 19, alkaline phosphatase on day 21, and protein per cell on day 23.

To grow *T. pseudonana* in semicontinuous culture for data shown in Fig. 7 (A to C), cultures were inoculated into various nutrient regimes and grown for 8 days before all cultures were diluted 50:50 with fresh ASW nutrient medium, and cultures were then diluted in the same manner every 2 days until experiments were performed on day 14. The alkaline phosphatase and phosphate uptake experiments (Fig. 7, D and E) were carried out on cultures inoculated into various nutrient regimes and cultured for 6 days; cultures grown in f/2 were diluted to 8 × 10^5^ cell/ml, low phosphate (0.225 μM) high nitrate (882 μM) to 4 × 10^5^ cell/ml, and colimited (0.225 μM phosphate, 5.5 μM nitrate) to 1.5 × 10^5^ cell/ml. This was repeated every 2 days until experiments were performed on day 10.

For the nutrient amendment experiments with both *P. tricornutum* and *T. pseudonana*, 4 × 200 μl of each biological replicate was removed from the main culture volume and added to a 96-well plate (catalog no. 83.3924, SARSTEDT). Nitrate (882 μM), phosphate (36 μM), or both depending on treatment were then added back to one well per biological replicate, with one well having no amendment. Growth was then monitored using chlorophyll fluorescence (RFU) at (excitation/emission) 440 ± 9/680 ± 20 nm wavelengths on a CLARIOstar Plus plate reader (BMG Labtech).

### Ca^2+^ imaging experiments using epifluorescence microscopy

*P. tricornutum* cells grown in various nutrient conditions were placed in a 35-mm glass-bottom dish (In Vitro Scientific) coated with 0.01% poly-l-lysine (Sigma-Aldrich). For the experiments using PtR1 grown in batch conditions, cells were imaged using a Nikon Eclipse Ti microscope with a 40×, 1.30 numerical aperture (NA) oil immersion objective and detection with a Photometrics Evolve EM-CCD camera (Photometric). To excite the R-GECO1 (PtR1) cells, a pE2 excitation system (CoolLED) was used with 530- to 555-nm excitation and 575- to 630-nm emission filters. NIS-ELEMENTS v.3.1 software (Nikon) was used to capture images with a 300-ms camera exposure. Because of equipment issues, the imaging of later experiments in which PtR1 cells were grown in semicontinuous conditions and the G-GECO1–mApple *P. tricornutum* line were carried out using a Leica DMi8 inverted microscope (Leica Microsystems) with a 63× 1.40 NA oil immersion objective. A SpectraX LED (light-emitting diode) light source (Lumencor) was used with a 550/15-nm excitation filter and 585/40-nm emission for R-GECO1 (PtR1). The different stimuli were applied using a gravity-fed perfusion system; in all cases, cells were initially perfused with ASW (pH 8.1) for 30 s. For the P-Ca^2+^ experiments with PtR1, the perfusion was then switched to use ASW + 36 μM Pi for 30 s before switching to 80% ASW (ASW diluted with distilled water) for another 30 s. To ensure that changes in R-GECO1 fluorescence intensity following phosphate resupply are not an artifact of alterations in intracellular pH, another Ca^2+^ biosensor line G-GECO1–mApple (*89*) was perfused with NH_4_Cl (10 mM) to trigger increases in internal pH. As both G-GECO1 and mApple exhibit mild sensitivity to pH but only G-GECO1 is sensitive to Ca^2+^, ratiometric (G-GECO1/mApple) imaging enables pH-driven effects to be distinguished from changes in cytosolic [Ca^2+^]. Increases in fluorescence intensity following phosphate resupply to P-limited cells far exceeded those detected upon NH_4_Cl exposure (fig. S12), confirming that they are a result of increases in cytosolic Ca^2+^ rather than pH-driven effects. For the G-GECO1–mApple line, the same setup as R-GECO1 was used to visualize mApple, and G-GECO1 used a 470/24-nm excitation filter and 525/50-nm emission filter, in a sequential manner. For the these experiments, G-GECO1–mApple cells were perfused for 30 s with ASW before exposure to 10 mM NH_4_Cl for 1 min before switching back to ASW for 30 s or exposed to ASW + 36 μM Pi for 1 min. Images were processed using either the NIS-ELEMENTS v3.1 or LasX (Leica) software for measurements made via the Nikon Eclipse Ti and Leica DMi8 microscopes, respectively. The mean fluorescence intensity (*F*) within a region of interest (ROI) drawn around each cell was measured over time. A background ROI mean fluorescence intensity was subtracted from each cellular *F* value. For PtR1 batch culture experiments, the fluorescence intensity was normalized by dividing the fluorescence intensity in each frame by the initial value (*F*/*F*_0_), and for the semicontinuous PtR1 experiments, the mean fluorescence of the six frames preceding the first stimuli were used to normalize all other frames due to noise in the signal. As the G-GECO1–mApple line can be used as a ratiometric Ca^2+^ indicator, the ratio was determined after the initial background ROI fluorescence was removed from each channel, then the G-GECO1/mApple ratio was calculated, and this was then normalized by using the average ratio of the first six frames (*R*/*R*_0_). Cells were deemed to have responded if *F*/*F*_0_ was greater than 1.15 [a threshold previously defined in (*56*)].

### Quantification of alkaline phosphatase activity

Alkaline phosphatase activity was quantified using the p-nitrophenyl phosphate (pNPP) liquid substrate system (Sigma-Aldrich, N7653). The protocol was modified from (*90*) and is based on the cleavage of pNPP by alkaline phosphatase resulting in a yellow substance. Briefly, cell-associated alkaline phosphatase was sampled by centrifuging a known volume of culture (1 to 3 ml) at 8000*g* for 10 min, removal of 70% of the supernatant, and centrifuging again (10,000*g*, 5 min). Cells were resuspended in 176.9 μl of alkaline phosphatase buffer [0.01 M tris (pH 8), 0.05 M MgCl_2_, and 0.01 M CaCl_2_] and transferred to 96-well plates. A total of 23.1 μl of pNPP liquid substrate was added to all sample wells, and alkaline phosphatase buffer with pNPP liquid substrate was used as a blank. The absorbance at 405 nm was measured at 2-min intervals for 30 min with an initial 10-min pause before first reading using a CLARIOstar Plus plate reader (BMG Labtech). Enzymatic activity was calculated using the change in absorbance over time, where the concentration = ∆absorbance at 405 nm/(path length × extinction coefficient). The extinction coefficient used was 18,000 M^−1^ cm^−1^, and the path length was 0.57 cm. The value was then normalized to the cell density.

### Phosphate uptake assay

To perform phosphate uptake experiments with *P. tricornutum*, 4-day-old batch cultures were used, and cell density was normalized to 2 × 10^6^ cell/ml. For the semicontinuous cultures, a cell count was performed to control for cell density differences. For the *T. pseudonana* experiments, cultures had been maintained for 10 days in a semicontinuous manner (detailed previously), and cell density was normalized to 6 × 10^5^ cell/ml. After normalizing cell densities, the colimited treatments were split to compare the effect of nitrate resupply on phosphate uptake. Nitrate was resupplied at the f/2 level of 882 μM, and cultures were incubated between 4 and 6 hours for *P. tricornutum* and 5 hours for *T. pseudonana*. To quantify phosphate uptake, a media sample was first taken to confirm the initial level of phosphate in the medium, 500 μl of sample was removed and centrifuged (10,000*g*, 5 min), and 200 μl of the supernatant was retained for analysis. Phosphate was then resupplied to cultures, and 500 μl of the sample was centrifuged (10,000*g*, 5 min), and 200 μl of the supernatant was retained for analysis at various increments between 0 and 180 min. This was repeated 24 hours later on colimited cultures with and without nitrate resupply, and cell density was counted to allow the calculation of rate of uptake per cell. Phosphate in the medium was quantified using the BIOMOL Green kit (Enzo Life Sciences) as per the manufacturer’s instructions, with three biological replicates and samples run in duplicate. Briefly, a standard curve was generated using the provided standard solutions, with ASW as the assay buffer. Fifty microliters of the sample was used, with 100 μl of BIOMOL GREEN reagent added to each sample. The 96-well plate was incubated at room temperature for 30 min, and the absorbance at 620 nm was measured using the CLARIOstar Plus plate reader (BMG Labtech). The rate of phosphate uptake was calculated by subtracting the phosphate detected in the media from 36 μM at the different time points and calculating the change in phosphate in the cell fraction per min. This was then normalized to the cell density to get picomolars per cell per minute. In the case of the *P. tricornutum* grown in semicontinuous conditions (fig. S5), cell density was not normalized before the experiment, but cell counts allowed the same calculations to be performed.

### Photosynthetic efficiency (*F*_v_/*F*_m_)

For quantifying photosynthetic efficiency of photosystem II (*F*_v_/*F*_m_), 2 ml of culture was first dark-adapted for 30 min. The default QY (quantum yield) parameter was then measured using an AquaPen-C device (Photon Systems Instruments).

### N uptake assay

In a similar fashion to the phosphate uptake experiments, *P. tricornutum* cells were grown in f/2, high nitrate low phosphate, low nitrate high phosphate, and low nitrate low phosphate conditions for 4 days in quadruplicate. Cell density was counted and concentrated to ~8 × 10^6^ cell/ml, and because of differences in the culture propensity to a centrifuge well, cell counts were also performed after this process. Cells were resuspended in ASW, and 300 μM nitrate was resupplied to each sample. After 10 min, the first sample was taken by filtering the culture through a 0.2-μm syringe top filter to remove the cells, the supernatant was retained for later analysis, and samples were frozen at −20°C. This was repeated after 1-, 3-, 24-, and 50-hour incubation, in standard growth conditions as described earlier. To quantify the amount of the nitrate remaining in the medium, the Nitrate/Nitrite Colorimetric Assay Kit (Cayman Chemical, 780001) was used according to the manufacturer’s recommendations for culture medium samples. Samples had to be diluted in the provided assay buffer to ensure they fell within the standard curve range generated. Further, all samples and standards were run in duplicate as recommended. The rate of uptake was then calculated by subtracting the amount of nitrate remaining in the culture medium from 300 μM to infer what had been taken up by the cell fraction. The change in nitrate per cell was then calculated as the rate of uptake in picomolars per cell per minute. As the low nitrate high phosphate and colimited cells removed all the N within 24 hours, the rate was calculated between 1 and 3 hours; for the f/2 grown cultures, between 3 and 24 hours was used, and for the high nitrate low phosphate cultures, 3 and 50 hours was used.

### PtPSR1-mVenus reporter line

The PtPSR1-mVenus reporter line was generated by synthesizing and custom cloning of a 2520-bp sequence (gene ID 47256) by GenScript (GenScript, Piscataway, NJ) into a derivative of the pPHA-T1 vector (accession AF219942), pPHA-T1-mVenus as described previously in (*88*). The synthesized fragment contains the gene sequence, including introns, as well as a 696-bp region upstream of the start codon (ATG), but excludes the stop codon. This construct was then transformed into *P. tricornutum* by biolistic bombardment, as previously described in (*88*) with zeocin (75 μg/ml) used for selection. In addition, the control strain expressing mVenus under the light-driven *fcpB* promotor was generated in the same way.

The transgenic PtPSR1-mVenus reporter line was grown in various media regimes as detailed previously and imaged using the LEICA SP8 inverted confocal microscope with a 63× 1.40 oil immersion objective. For mVenus fluorescence, an excitation wavelength of 488 nm and emission between 506 and 556 nm were used. Chlorophyll fluorescence was detected using the excitation wavelength of 561 nm and an emission of 649 to 750 nm. To investigate how N level changed the expression of PtPSR1-mVenus expression, cultures were grown in f/2 media and low phosphate (1.8 μM) varying nitrate (882, 44, and 22 μM) regimes for 4 days in 24-well plates. The fluorescence level was measured using the CLARIOstar Plus plate reader (BMG Labtech) using the following wavelengths (excitation/emission): 515 ± 10/550 ± 10 nm (mVenus, RFU) and 440 ± 9/680 ± 20 nm (chlorophyll, RFU). The mVenus fluorescence was then normalized to chlorophyll fluorescence to account for cell density differences for experiments shown in Fig. 5B.

To investigate the effect of N resupply on PtPSR1-mVenus expression in colimited cells, cultures were grown in triplicate for 4 days as previously described, a sample of each culture was imaged as above and then the colimited cultures split, and nitrate was resupplied (882 μM) to half of the samples. The colimited cells with and without N resupply were imaged 4 and 10 hours later, and all nutrient regimes were imaged 24 hours later. Images were processed using Fiji (*91*) with ROIs drawn around the bright-field image of at least eight cells per replicate and then used as ROIs on the mVenus channel image to obtain the mean intensity per cell.

### Transformation and Ca^2+^ imaging of *Ptpsr1* mutants expressing R-GECO1-mTurquoise

The WT *P. tricornutum* strain was transformed with RGECO1-mTurquoise_pPHAT1 plasmid and selected using zeocin (75 μg/ml). The *Ptpsr1* mutants were transformed with RGECO1-mTurquoise_pPTbsr and selected using blasticidin (8 μg/ml) using the methods detailed in (*88*).

Cells were grown in six-well plates in a 6-ml volume of f/2 or low phosphate (1.8 μM) medium. Cells were imaged using a Leica DMi8 inverted microscope (Leica Microsystems) with a 63× 1.40 NA oil immersion objective. A SpectraX LED light source (Lumencor) was used with a 550/15-nm excitation filter and 585/40-nm emission filter for R-GECO1 and a 470/24-nm excitation filter and a 525/50-nm emission filter for mTurquoise in a sequential manner. As previously detailed, a gravity-fed perfusion system was used, with ASW (pH 8.1) perfused for 25 s before switching to ASW + Pi (36 μM). As the R-GECO1–mTurquoise line can be used as a ratiometric Ca^2+^ indicator, the initial background ROI fluorescence was removed from each channel, then the R-GECO1/mTurquoise ratio was calculated, and this was then normalized by using the average ratio of the six frames preceding the Pi resupply stimulus (*R*/*R*_0_).

### Protein content

*P. tricornutum* cells were grown in semicontinuous culture; cell counts were performed; and for three replicates of the low phosphate low nitrate cultures, nitrate was resupplied (882 μM). All cultures were harvested 6 hours later by centrifugation (3500*g*, 15 min). f/2 grown cultures were harvested in 50-ml aliquots and the low nutrient cultures in 100-ml aliquots and snap frozen in liquid nitrogen before storage at −80°C until further processing. On ice, a master mix of radioimmunoprecipitation assay lysis and extraction buffer (Thermo Fisher Scientific) with Halt Protease Inhibitor Cocktail (Thermo Fisher Scientific) at a ratio of 100:1 was prepared, and 200 μl was added to each sample aliquot. This was then transferred to liquid nitrogen–cooled pestles for manual grinding with liquid nitrogen. Samples were centrifuged in microfuge tubes for 10 min at 17,000*g*, 4°C, and the supernatant was transferred to new tubes, with the low nutrient aliquots pooled so a total of 200 ml of culture was used per replicate and 100 ml of f/2 grown cultures. This was then used to perform the Pierce BCA Protein assay (Thermo Fisher Scientific) according to the manufacturer’s instructions for microplates to quantify the protein level. The protein content per cell was than calculated.

### Statistical analysis

Figures were generated, and analyses were performed using R statistical software [v4.2.2; (*92*)]. The R package “ggpubr” was used for analyzing the Ca^2+^ signaling data (*93*). Other data were tested for normality, and either the corresponding parametric or nonparametric test was applied. The Dunn post hoc test was carried out using the R package “FSA” (*94*).
