# Supplementary material for: Nitrogen status exerts dynamic control over phosphorus sensing and acquisition via PSR1 in colimited marine diatoms
Source: Sci Adv. 2025 Aug 29;11(35):eadw8260. doi: 10.1126/sciadv.adw8260 (PMC12396336; doi:10.1126/sciadv.adw8260)
Supplement: Supplementary file 1 — Figs. S1 to S12 [file sciadv.adw8260_sm.pdf]

Science Advances
AAAS

Supplementary Materials for

Nitrogen status exerts dynamic control over phosphorus sensing and acquisition via PSR1 in colimited marine diatoms

Ellen Harrison *et al.*

Corresponding author: Katherine Helliwell, k.helliwell@exeter.ac.uk, katherine.helliwell@mba.ac.uk

Sci. Adv. **11**, eadw8260 (2025)
DOI: 10.1126/sciadv.adw8260

This PDF file includes:

Figs. S1 to S12

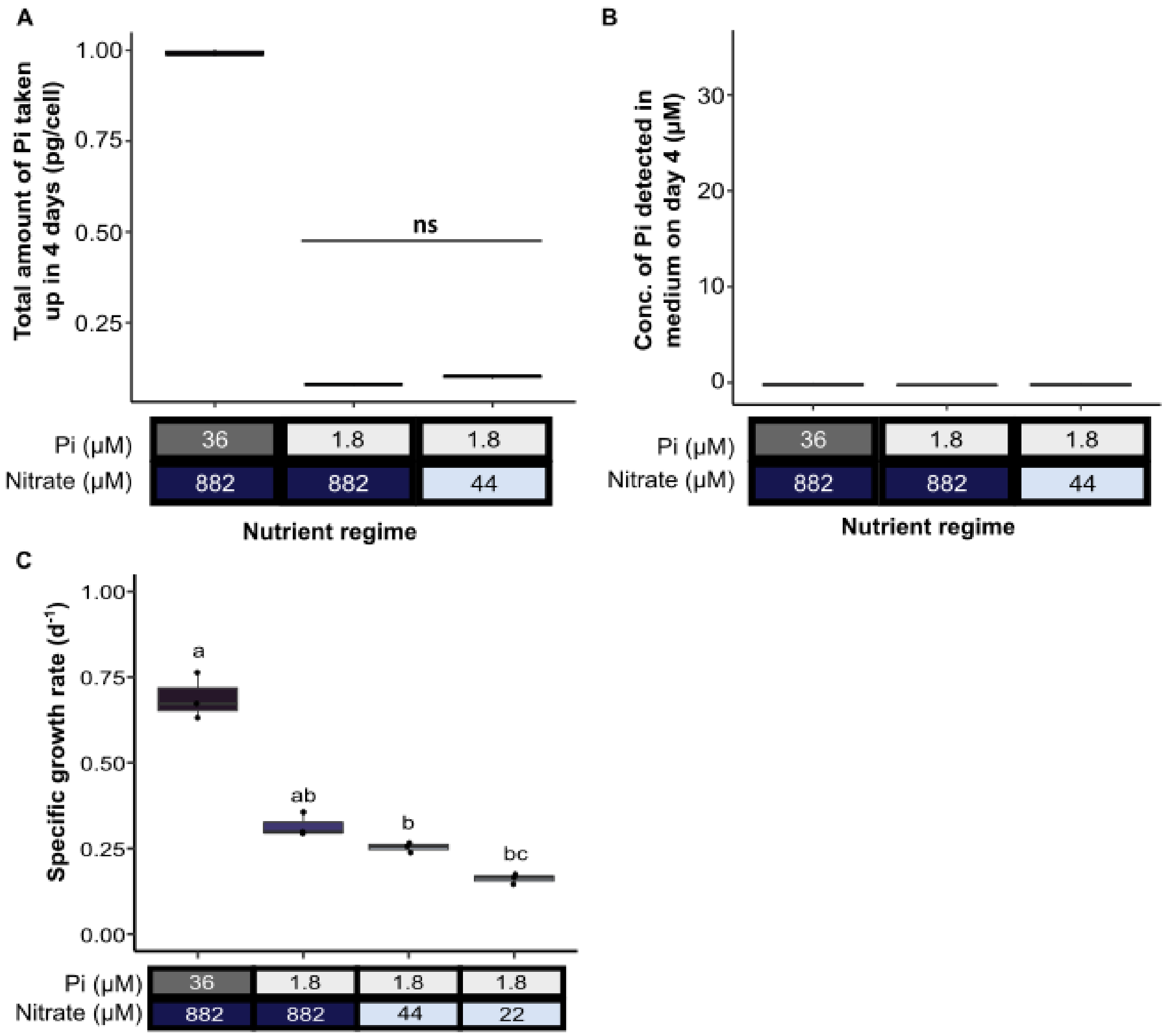

Fig. S1.

***Phaeodactylum tricornutum* removes phosphate (Pi) from medium within 4-days and the resulting total amount of Pi taken up per cell. A)** Pi taken up per cell (pg per cell) after 4-days in replete, P-limited and low N low P treatments. Dunn post-hoc test showed no significant (ns) difference between cells grown at low phosphate (1.8 µM) high nitrate (882 µM) and low phosphate low nitrate (44 µM) cultures (*n*=3). **B)** Confirmation that all the Pi in the initial medium had been taken up by day 4 of the experiment, measuring the concentration of Pi in the external medium using a BIOMOL® Green assay (*n*=3). **C)** Representative specific growth rate (d^{-1}) of batch cultures used in *P. tricornutum* experiments. (*n*=3, letters indicate statistically significant groupings based on Dunn post-hoc test, *p*-values<0.05).

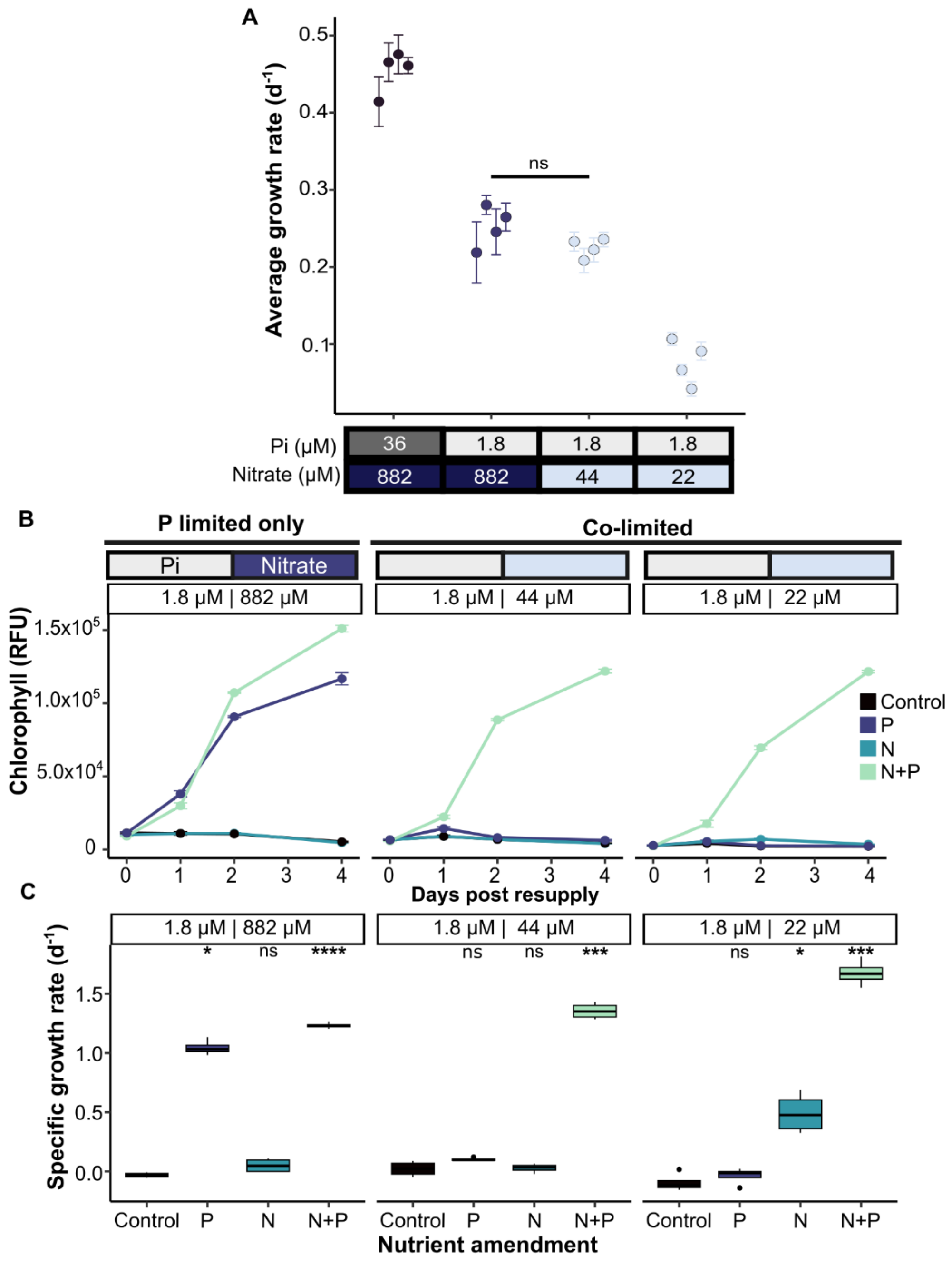

Fig. S2.

Confirmation of co-limitation by nutrient amendment experiments in semi-continuously grown *Phaeodactylum tricornutum*. A) Average growth rate (d^{-1}) per biological replicate (n=4) from day 4 onwards (over 5 dilution events, ±SD). Line indicates result of Dunn post-hoc test, all other comparisons significantly different (p-values ≤0.001). **B**) Chlorophyll fluorescence as a proxy for growth of nutrient amendment experiment, cultures grown in semi-continuous culture for 15 days with sub-culturing occurring every 2-3 days before a sample was taken and grown with either the addition of Pi (P), nitrate (N), Pi and nitrate (N+P) or with no supplementation ('control')

(*n*=3 ±SEM).C) Specific growth rate calculated between day 0 and 2 following amendment treatments, asterisks indicate result of Dunn post-hoc test compared to the control with *p*-values described as follows: $p < 0.05$; *, $p < 0.01$; ***, $p < 0.001$ ****, ‘ns’ indicates the result was not significant (*n*=3).

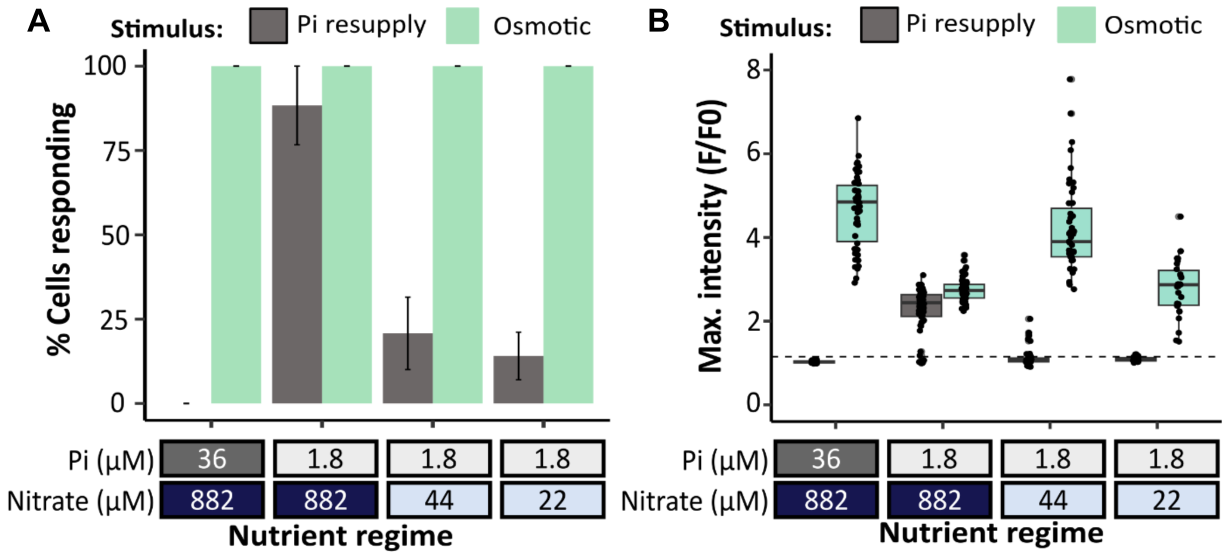

Fig. S3.

P and N co-limited *Phaeodactylum tricornutum* cells grown in semi-continuous culture show a reduced capacity for P-Ca^{2+} signalling. A) Percentage of *P. tricornutum* PtR1 cells that respond to 36 µM phosphate resupply or an 80% artificial seawater (ASW) osmotic shock, with at least 8 cells per biological replicate analysed. Error bars indicate the SEM of 3 replicates. **B)** The maximal fluorescence (F/F0) of all cells analysed to Pi resupply or osmotic shock, 1.15 threshold indicated by a dashed horizontal line.

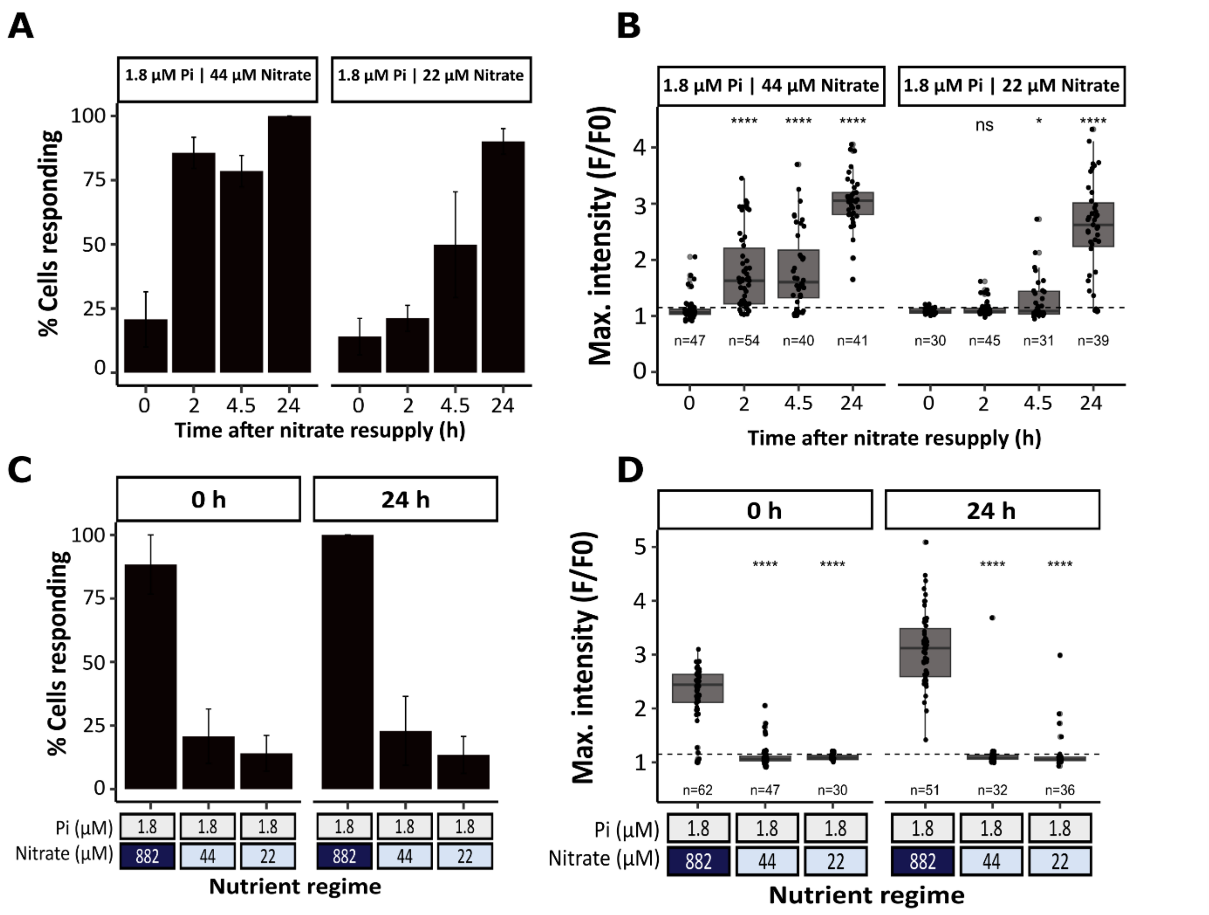

Fig. S4.

P-Ca^{2+} signalling response is restored within 2 h following nitrate resupply to N and Pi co-limited cells grown semi-continuously. A) Time-course following nitrate resupply to semi-continuously N and P co-limited cultures grown either in low Pi (1.8 µM) and 44 or 22 µM nitrate, showing the percentage of cells which respond to a Pi resupply (36 µM), using a max. fluorescence (F/F0) of 1.15 as a threshold. Experiment performed on three sets of independently grown cultures per treatment, with at least 8 cells per replicate analysed. Error bars shows the SEM of 3 replicates. **B)** The maximal fluorescence (F/F0) to Pi resupply of cells during the experimental time-course. Horizontal line indicates 1.15 F/F0 threshold. The control experiments, showing the percentage of cells which respond to Pi resupply when grown in low Pi (1.8 µM) high nitrate (882 µM) and the two co-limited conditions at timepoint 0 and 24 h later without nitrate resupply (**C**), and the corresponding F/F0 (**D**). Asterisks indicate Student's *t*-test result compared to timepoint 0 (**B**) or to the P-limited only control (**D**) with *p*-values described as follows: $p < 0.05$; *, $p < 0.01$; ***, $p < 0.001$ ****, 'ns' indicates the result was not significant.

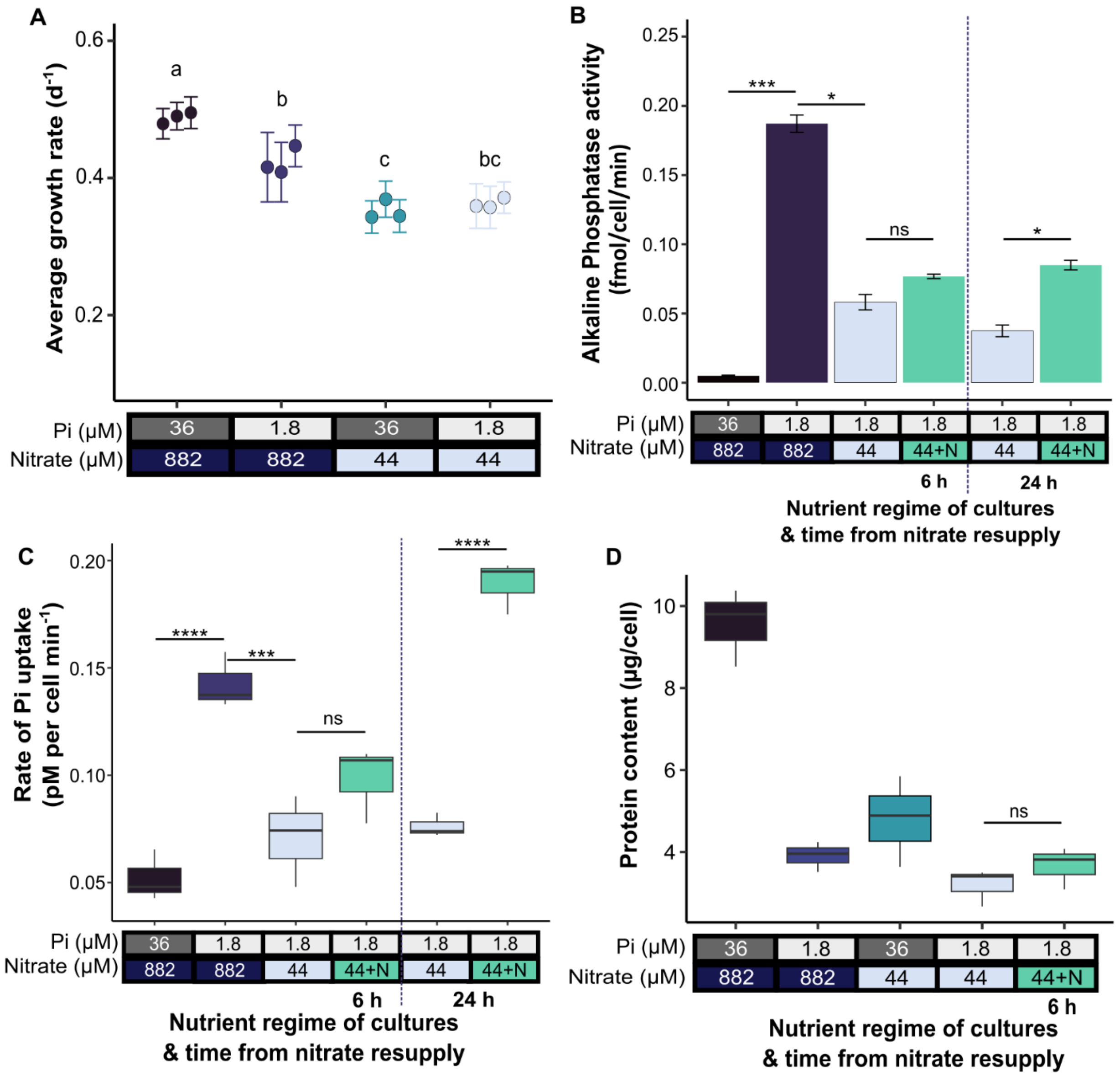

Fig. S5.

Confirmation of N status governing P scavenging and acquisition in semi-continuous *P. tricornutum* cultures. A) Average growth rate (d^{-1}) per biological replicate (n=3) across sub-culturing dilutions from day 12 onwards of cells grown in f/2, low Pi (1.8 µM) high nitrate (882 µM), low nitrate (44 µM) high Pi (36 µM) and low Pi (1.8 µM) low nitrate (44 µM), averaged across 5 sub-culturing events (±SEM). Letters indicate statistically significant groups (Dunn post-hoc test, p-value≤0.05) **B)** Alkaline phosphatase activity (fmol/cell/min) of cells grown in f/2, low Pi (1.8 µM) high nitrate (882 µM), low Pi (1.8 µM) low nitrate (44 µM) or low Pi low nitrate resupplied with 882 µM nitrate, 6 h and 24 h post nitrate resupply (n=3, ±SEM), vertical dashed line to highlight that measurement was repeated on those samples 24 h later. Lines indicate the comparison and asterisks the result of Dunn post-hoc test, p-values described as follows: $*p < 0.05$; $**p < 0.01$; $***p < 0.001$, $****p < 0.0001$, 'ns' indicates the result was not significant. **C)** Rate of Pi uptake (pM per cell min^{-1}) calculated between 30-80 mins after Pi resupply (n=3), vertical dashed line to highlight that experiment was repeated on those samples 24 h later. Lines

indicate the comparison and asterisks the result of Tukey post-hoc test, *p*-values as described in **B**. **D)** Protein content per cell depending on nutrient regime, nitrate was resupplied to co-limited cultures and all treatments harvested after 6 h, *n*=3. Dunn post-hoc test showed no significant (ns) difference between co-limited condition and with nitrate resupplied.

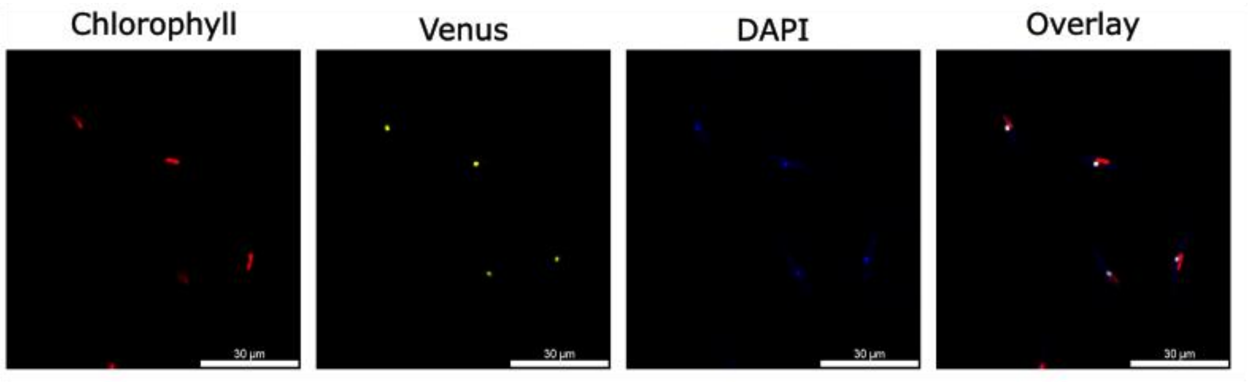

Fig. S6.

Localisation of PtPSR1-mVenus to the nucleus. Localisation of PtPSR1-mVenus in *Phaeodactylum tricornutum* grown in low Pi (1.8 µM) high nitrate (882 µM) medium and imaged using confocal microscopy stained with DAPI to confirm nuclear localisation, scale bar: 30 µm.

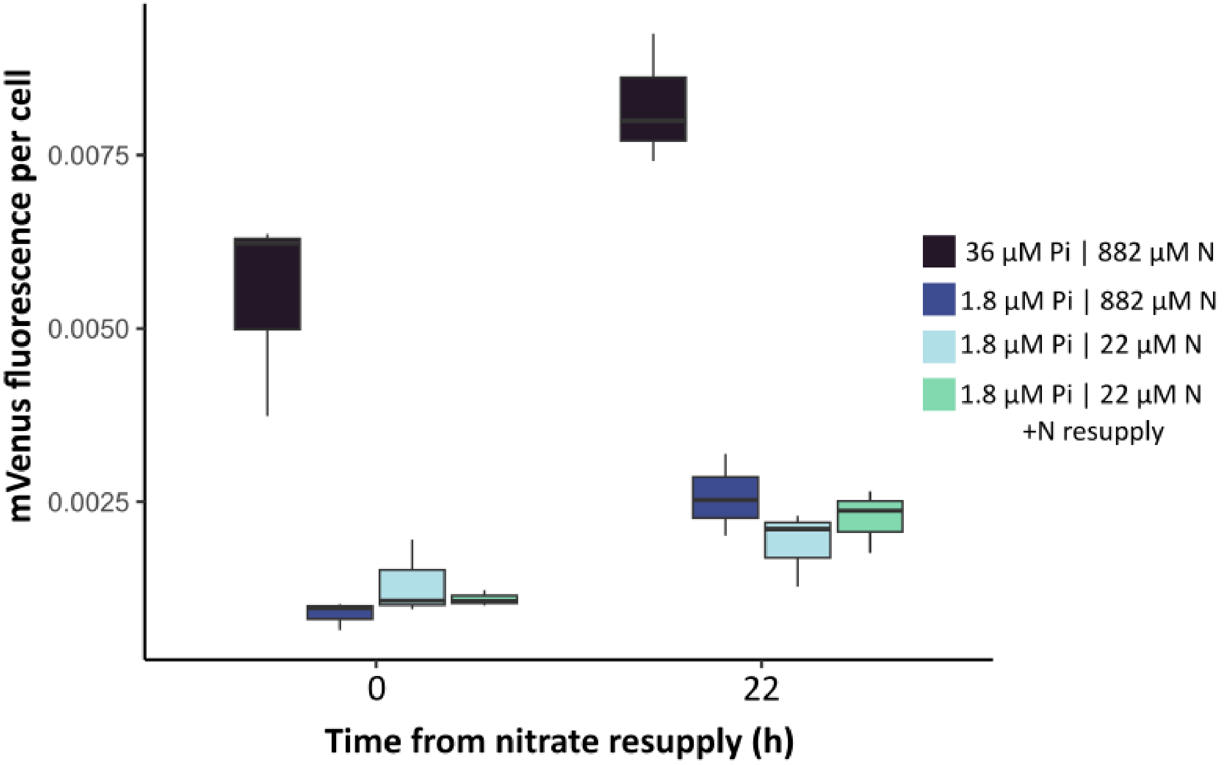

Fig. S7.

Expression of mVenus protein under the light-responsive *fcpB* promotor in different nutrient regimes. mVenus fluorescent protein expressed by *P. tricornutum* under the *fcpB* light-driven promotor when grown in f/2, low Pi (1.8 µM) high nitrate (882 µM) and low Pi (1.8 µM) low nitrate (22 µM) with and without N resupply (+882 µM nitrate), fluorescence (relative fluorescence unit, RFU) is normalised to the cell count, n=3.

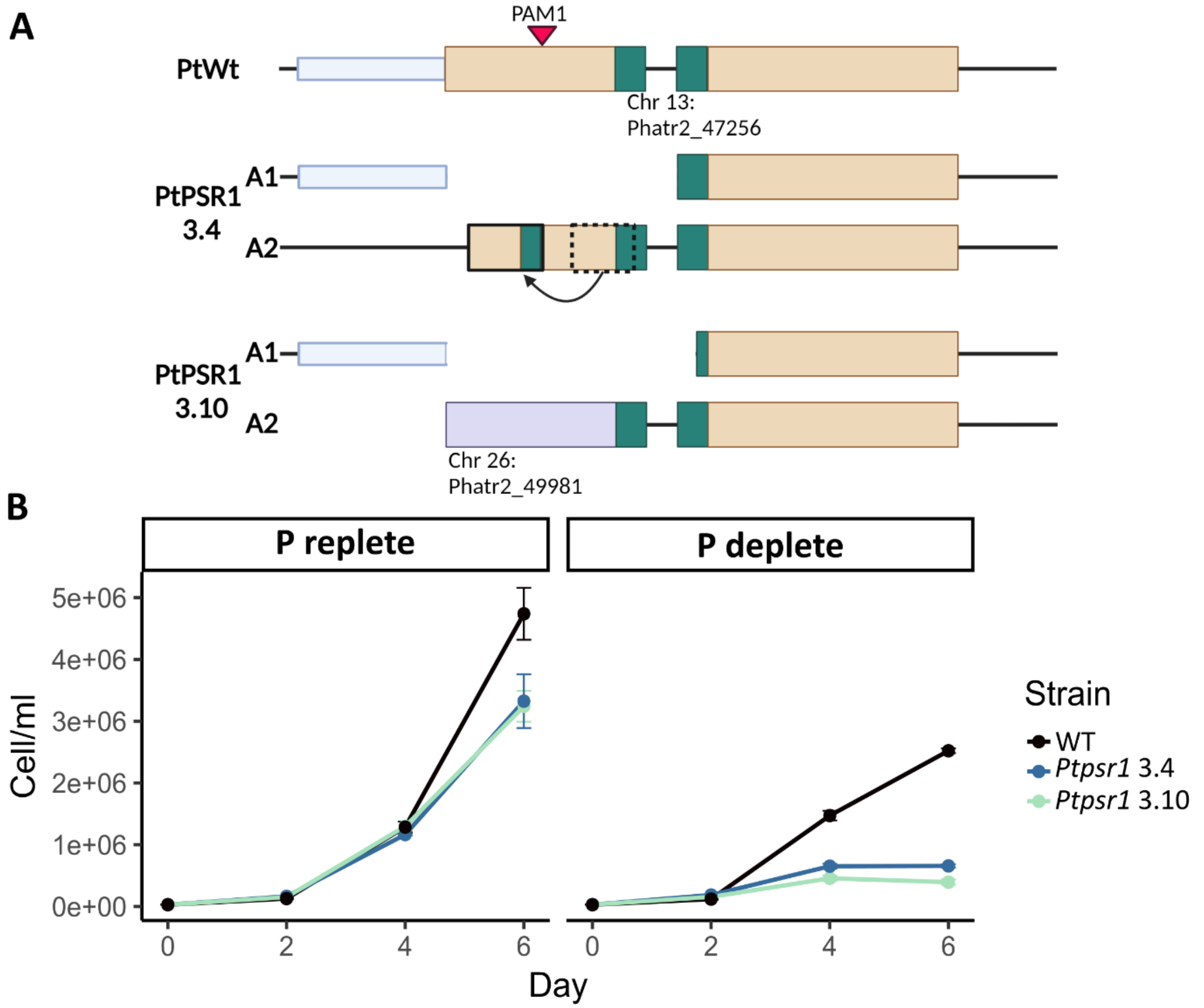

Fig. S8.

Summary of *Ptpsr1* mutants with Ca^{2+} biosensor. A) Schematic representation of rearrangements and deletions in *Ptpsr1* mutants compared to wildtype, redrawn from Sharma *et al.* (*36*). Disruptions in the nucleotide sequence for allele 1 (A1) and allele 2 (A2) of the gene are shown. The protospacer adjacent motif (PAM) 1 site for the sgRNA target is also indicated. Green indicates region encoding the Myb domain, brown shows exons, insertion of new region shown in purple, dashed box and arrow indicate tandem duplication. **B)** Growth of R-GECO1-mTurquiose lines in wild-type (WT) and *Ptpsr1* mutant backgrounds in replete (36 µM) phosphate and P deplete (1.8 µM) medium, *n*=3 ±SEM.

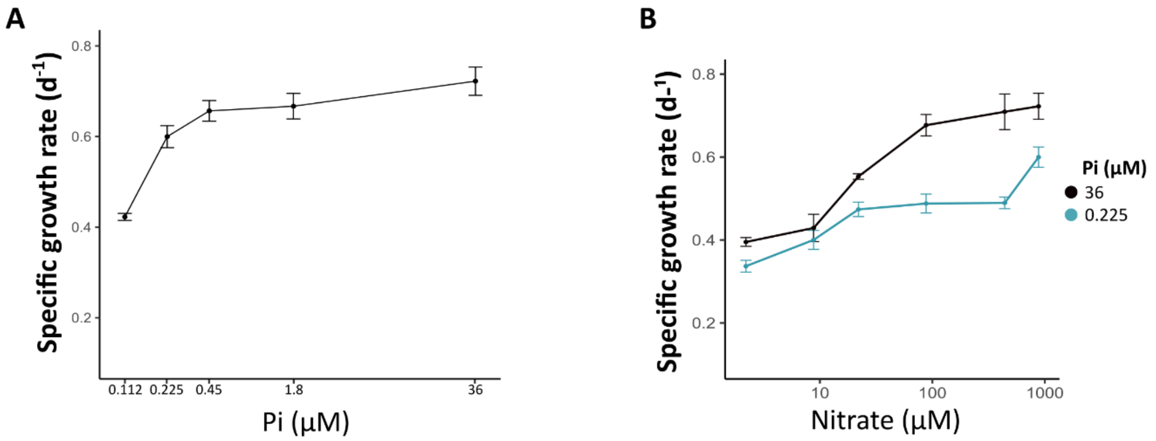

Fig. S9.

Defining N and P co-limiting conditions for *Thalassiosira pseudonana*. A) Specific growth rate (d^{-1}) of *T. pseudonana* (strain CCMP 1335, Material and Methods) grown on f/2 medium with different concentrations of phosphate (Pi): 0.112, 0.225, 0.45, 1.8, 36 µM. Growth rate was calculated during exponential growth phase, between 2 and 4 days (*n*=4 ±SEM). **B)** As in (**A**) but cultures were grown on one of two Pi concentrations (36 µM in navy, 0.225 µM in light blue), but with various levels of nitrate, ranging from 2.2 to 882 µM (*n*=4 ±SEM).

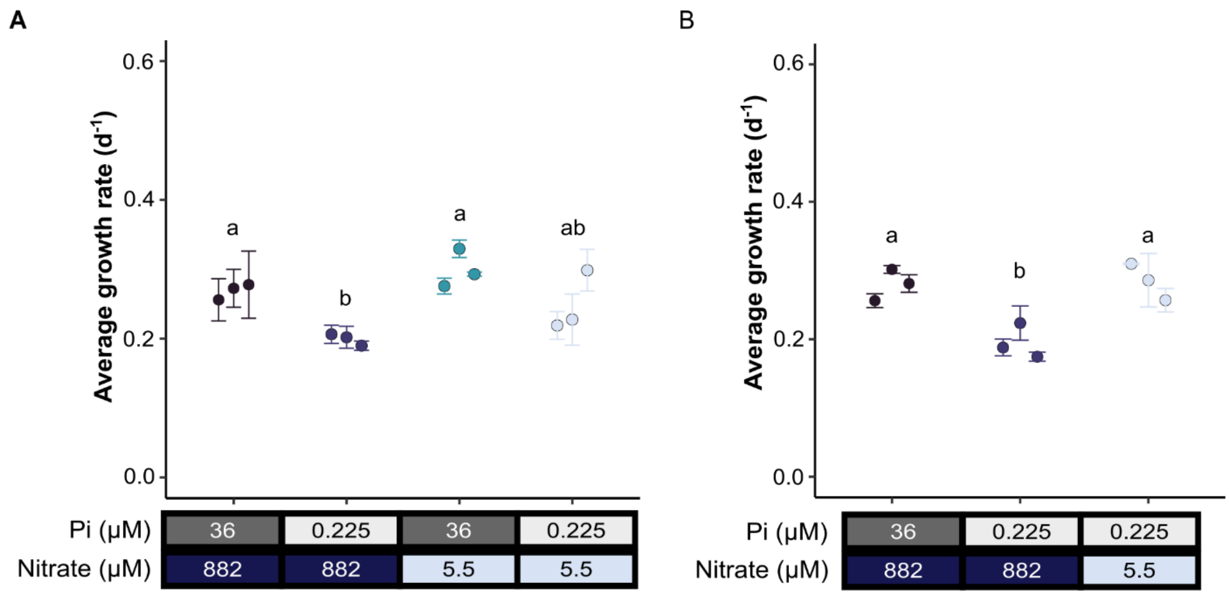

Fig. S10.

Average growth rate (d^{-1}) of *Thalassiosira pseudonana* grown in semi-continuous culture. A) Cultures grown in different nutrient regimes used in experiments shown in Fig. 7A-C, each point shows the average growth rate (d^{-1}) per biological replicate (n=3) for sub-culturing dilution events from day 8 onwards (averaged across 3 dilution events ±SEM). **B)** shows the average growth rate (d^{-1}) per replicate of cultures used in experiments shown in Fig. 7D-E, from day 6 onwards, averaged across two dilution events ±SEM. Letters indicate statistically significant groups (Tukey post-hoc test, p-value≤0.05).

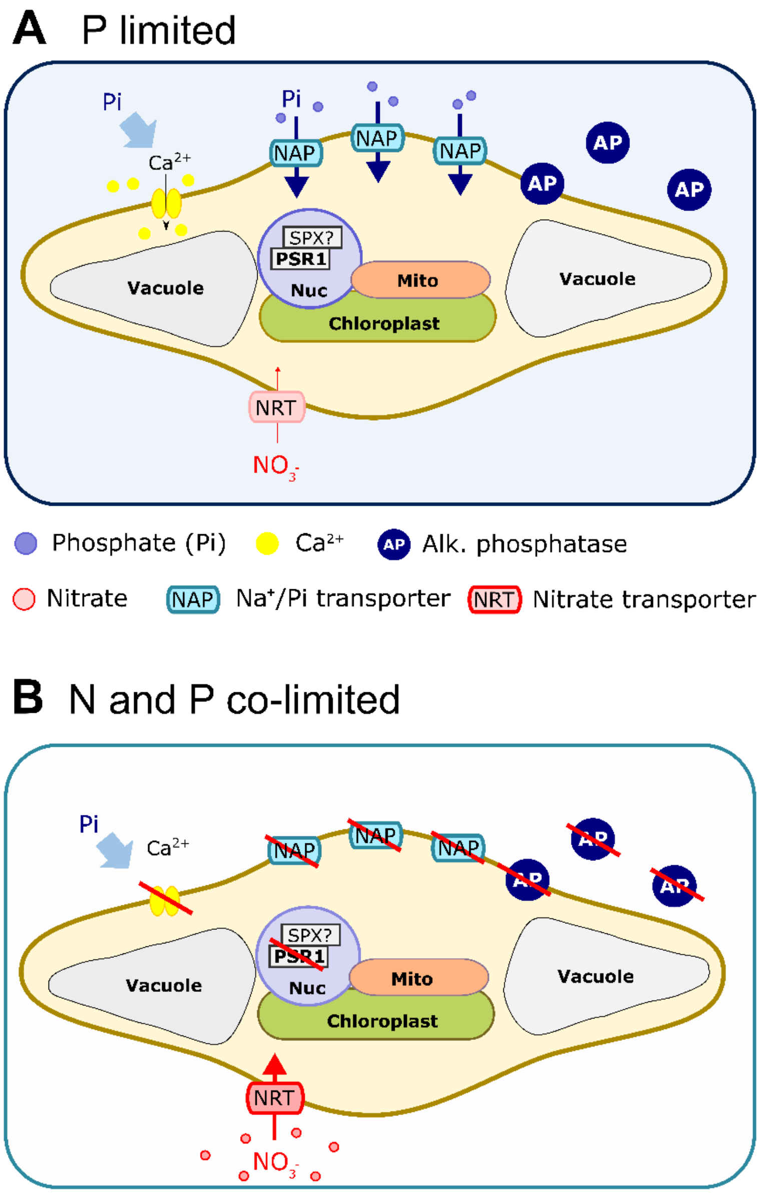

Fig. S11.
A model illustrating changes in *Phaeodactylum tricornutum* nutrient signalling and acquisition in P limited versus P and N co-limited cells. A) Under P limitation *P. tricornutum* enhances phosphate (Pi) uptake via Na$^+$/Pi transporters and alkaline phosphatase expression (*22*, *23*, *33*, *35*) coordinated by PtPSR1 (*36*). Cells also detect Pi resupply via P-Ca^{2+} signalling (*37*). N uptake by comparison is reduced in P-limited cells compared to replete conditions. **B)** In co-limiting conditions, P starvation coping mechanisms including P signalling and upregulated acquisition machinery are de-prioritised, with N uptake instead promoted.

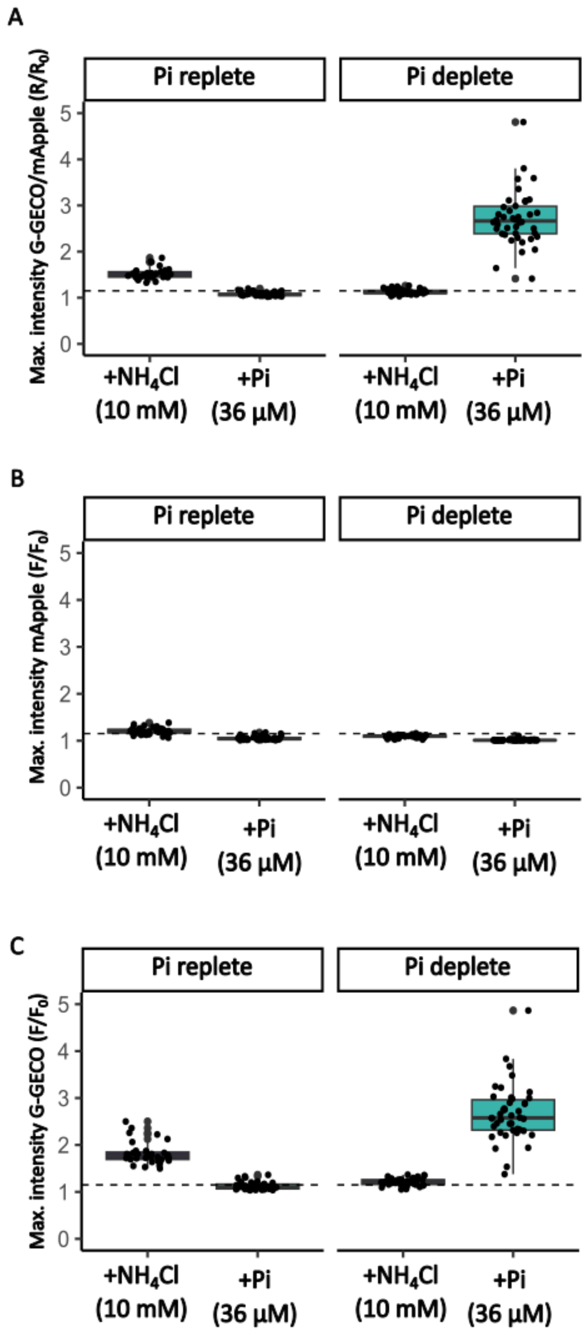

Fig. S12.
Phosphate induced increases in Ca^{2+} biosensor fluorescence in P-limited *Phaeodactylum tricornutum* cells are driven by rises in cytosolic Ca^{2+} rather than pH. A) Box plots showing maximal change in G-GECO1-mApple fluorescence ratio (R/R0) of cells grown in Pi replete (36 µM) or Pi deplete (1.8 µM) medium for 4 days, in response to 10 mM NH_4Cl that induces strong cytosolic alkalinization or 36 µM Pi resupply. The experiment was performed on 3 independent replicates with at least 7 cells analysed per replicate. Changes in maximal fluorescence intensity (F/F0) of mApple (**B**) and G-GECO1 (**C**) individually in response to the described treatments are also shown.
